# Mutation in a PHD-finger protein MS4 causes male sterility in soybean

**DOI:** 10.1186/s12870-019-1979-4

**Published:** 2019-08-28

**Authors:** Sandi Win Thu, Krishan Mohan Rai, Devinder Sandhu, Alex Rajangam, Vimal Kumar Balasubramanian, Reid G. Palmer, Venugopal Mendu

**Affiliations:** 1 Fiber and Biopolymer Research Institute, Department of Plant and Soil Science, Texas Tech University, Lubbock, TX 79409 USA; 2 US Salinity Laboratory (USDA-ARS), Riverside, CA 92507 USA; 3 Wisconsin Institute of Sustainable Technology, University of Wisconsin-Stevens Point, Stevens Point, WI 54481 USA; 4 Department of Agronomy, Iowa State University, Ames, IA 50011 USA

**Keywords:** Fertility, Mapping, *mmd1*, *ms4*, Plant homeodomain, Soybean, Sterility

## Abstract

**Background:**

Male sterility has tremendous scientific and economic importance in hybrid seed production. Identification and characterization of a stable male sterility gene will be highly beneficial for making hybrid seed production economically feasible. In soybean, eleven male-sterile, female-fertile mutant lines (*ms1, ms2, ms3, ms4, ms5, ms6, ms7, ms8, ms9, msMOS,* and *msp*) have been identified and mapped onto various soybean chromosomes, however the causal genes responsible for male sterility are not isolated. The objective of this study was to identify and functionally characterize the gene responsible for the male sterility in the *ms4* mutant.

**Results:**

The *ms4* locus was fine mapped to a 216 kb region, which contains 23 protein-coding genes including *Glyma.02G243200*, an ortholog of Arabidopsis *MALE MEIOCYTE DEATH 1* (*MMD1*), which is a Plant Homeodomain (PHD) protein involved in male fertility. Isolation and sequencing of *Glyma.02G243200* from the *ms4* mutant line showed a single base insertion in the 3rd exon causing a premature stop codon resulting in truncated protein production. Phylogenetic analysis showed presence of a homolog protein (MS4_homolog) encoded by the *Glyma.14G212300* gene. Both proteins were clustered within legume-specific clade of the phylogenetic tree and were likely the result of segmental duplication during the paleoploidization events in soybean. The comparative expression analysis of *Ms4* and *Ms4_homologs* across the soybean developmental and reproductive stages showed significantly higher expression of *Ms4* in early flowering (flower bud differentiation) stage than its homolog. The functional complementation of Arabidopsis *mmd1* mutant with the soybean *Ms4* gene produced normal stamens, successful tetrad formation, fertile pollens and viable seeds, whereas the *Ms4_homolog* was not able to restore male fertility.

**Conclusions:**

Overall, this is the first report, where map based cloning approach was employed to isolate and characterize a gene responsible for the male-sterile phenotype in soybean. Characterization of male sterility genes may facilitate the establishment of a stable male sterility system, highly desired for the viability of hybrid seed production in soybean. Additionally, translational genomics and genome editing technologies can be utilized to generate new male-sterile lines in other plant species.

**Electronic supplementary material:**

The online version of this article (10.1186/s12870-019-1979-4) contains supplementary material, which is available to authorized users.

## Highlight

Fine mapping of soybean *ms4* resulted in identification of male sterile phenotype causal mutation. The MS4 is a PHD-finger protein which is functionally characterized using Arabidopsis *mmd1* mutant.

## Background

Soybean (*Glycine max*) is one of the most important food crops in the world known for its high seed protein and oil content [1]. In general, the crop performance and yield can be increased by generating hybrid plants which outcompete pure lines due to heterosis or hybrid vigor [2]. As soybean is a self-pollinating crop, producing large quantities of hybrid seed by manual emasculation and cross-pollination is difficult and economically non-viable. Hence, identification of a stable male sterility system similar to rice and maize is much needed in soybean [3]. Male sterility resulting from mutations in genes involved in microsporogenesis and/or microgametogenesis has been described in various plant species [4]. Mutations in the genes involved in synapsis (proper chromosomal pairing and gamete formation) are known to be responsible for male-sterile female-sterile, male-sterile female-fertile or male-fertile female-sterile phenotypes in plants [5]. In soybean, so far, a total of 11 male-sterile, female-fertile (*ms1, ms2, ms3, ms4, ms5, ms6, ms7, ms8, ms9, msMOS and msp*) mutants have been identified and mapped on to different chromosomes [6–8]. Further, genetic inheritance and allelism tests, revealed that these 11 soybean male-sterile mutants are genetically independent of each other [7]. Cytological studies on these mutants suggested various mechanisms governing the male-sterile phenotype, which include cytokinesis failure during telophase II, failure of tetrad formation during microsporogenesis, microspore and pollen degeneration, abnormality/lack of vacuoles, and low callose level [4, 7, 9–14]. Molecular characterization of the male sterility genes will be the key to establish a sterility system for commercial application in hybrid seed production.

**Fig. 1:**
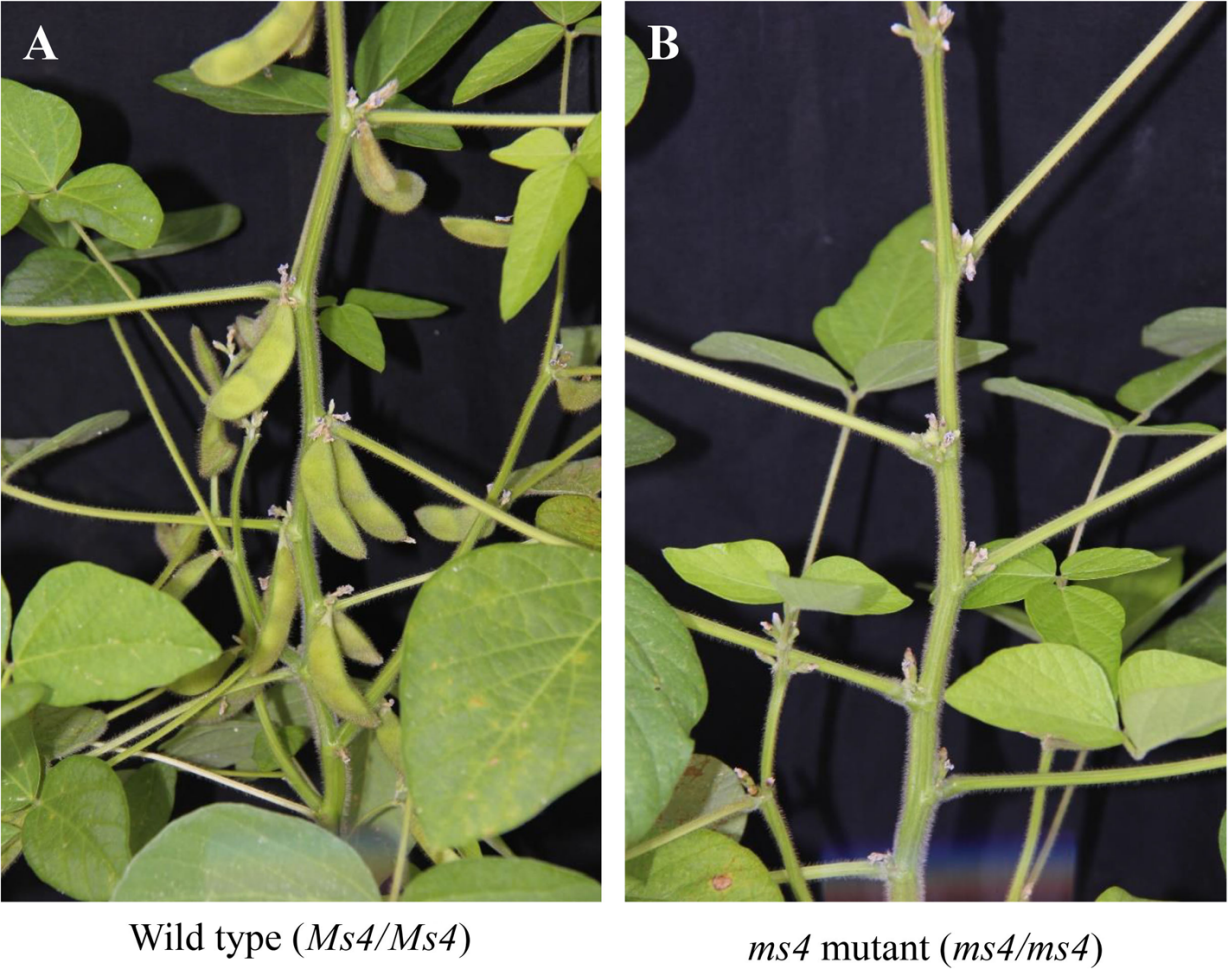
Phenotype of soybean wild type and the *ms4* mutant. (**a**) Wild type plant produces normal pods filled with seeds (**b**) The mutant plant produces sterile pollen resulting in no pod formation

Among the male-sterile mutant lines, the *ms4* mutant was an outcome of a spontaneous mutation in cultivar “Rampage” that was identified at the Iowa State University in 1973 (Fig.1) [15]. Phenotyping of homozygous *ms4/ms4* plants showed that the anthers were slightly smaller in size and lighter in color compared to the control [15]. The *ms4* mutant forms clumps of degenerated empty microspores due to the failure of cytokinesis after telophase II resulting in coenocytic microspores [10]. Genetic analysis of the *ms4* mutant line revealed that the male-sterile phenotype is governed by a single recessive gene that mapped to a 694 kb region containing 88 predicted protein-coding genes on soybean chromosome 2 [6]. Fine mapping and/or causal gene identification of *ms4* will help in designing molecular markers to screen male-sterile plants for hybrid seed production. The process of hybrid seed production requires planting pure male-sterile plants next to the donor soybean genotypes in field for cross pollination to occur. However, a male-sterile line cannot be maintained in homozygous condition as it does not produce seed, hence, is maintained in heterozygous condition. For hybrid seed production, male-fertile plants (homozygous and heterozygous) that arise in the progeny of heterozygous male-sterile plants need to be eliminated to prevent self-pollination. Identification of the causal male-sterility gene can facilitate marker-based genotyping of the segregating male-sterile population to eliminate fertile plants during the seedling stage before flowering. In addition, the functional characterization of male-sterile genes will also help to elucidate the molecular mechanism responsible for the male sterility and extend the technology to other crops to develop male-sterile lines using genetic engineering.

In the present investigation, we have fine mapped the *Ms4* locus to a 216 kb region containing 23 predicted protein-coding genes, identified the candidate gene (*Glyma.02G243200*) based on the functional relevance and characterized it using Arabidopsis model system. The protein sequence analysis showed that *Ms4* (*Glyma.02G243200*) is an ortholog of Arabidopsis *MALE MEIOCYTE DEATH 1* (*MMD1*), a PHD-finger protein involved in male fertility. Further, functional characterization of *Glyma.02G243200* using Arabidopsis *mmd1* mutant restored the male-fertile phenotype in Arabidopsis, thereby confirming its role in male fertility.

## Results

### Gene discovery by fine mapping of the *ms4* locus

**Fig. 2:**
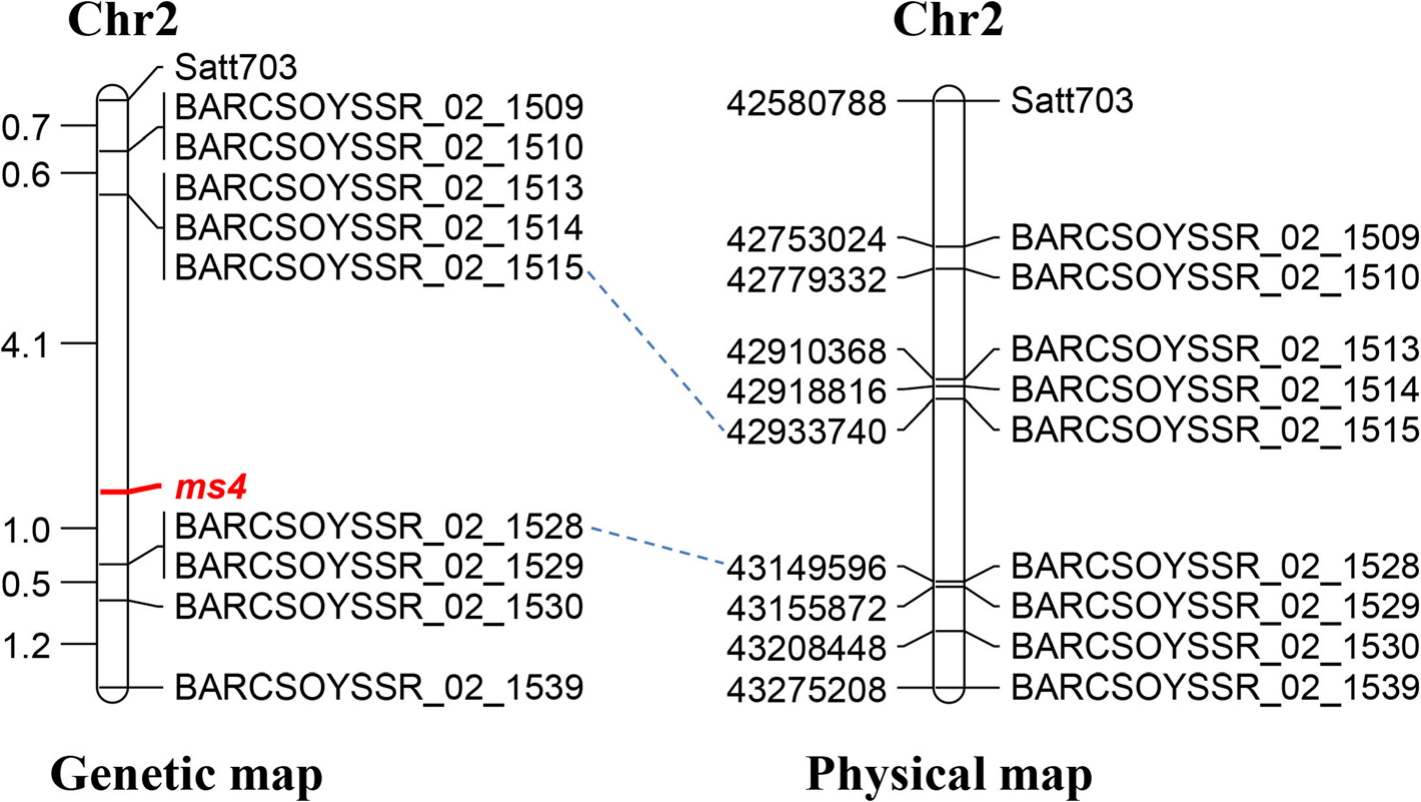
Maps of MLG D1b (Chromosome Gm02) showing location of *ms4*. The genetic linkage map and sequenced based physical map of the soybean chromosomes showing locations of SSR markers close to the *ms4* locus. Genetic distances are shown in centiMorgans whereas physical distances are shown in base pairs

We previously mapped the *ms4* locus on to chromosome 2 between the microsatellite markers Satt703 and BARCSOYSSR_02_1539 [6]. This region physically spanned 694 kb and harbored 88 predicted protein-coding genes. To further fine map the *ms4* locus, 20 potential SSR (Simple Sequence Repeat) markers were used to test the polymorphism in the chromosomal region between Satt703 and BARCSOYSSR_02_1539 of the parental lines of the mapping population; T274H (*ms4/ms4*) as a female parent and Manchu (*Ms4/Ms4*) as a male parent. Of these, 8 markers (BARCSOYSSR_02_1509, BARCSOYSSR_02_1510, BARCSOYSSR_02_1513, BARCSOYSSR_02_1514, BARCSOYSSR_02_1515, BARCSOYSSR_02_1528, BARCSOYSSR_02_1529 and BARCSOYSSR_02_1530) showed polymorphism between the two parental lines. These 8 polymorphic markers were further used for fine mapping of the *ms4* locus using an F_2_ population consisting of 118 plants. The *ms4* locus was mapped to 5.1 cM region between BARCSOYSSR_02_1515 and BARCSOYSSR_02_1528 (Fig. 2). The corresponding region on the soybean physical map was 216 kb with 23 predicted protein-coding genes (Fig. 2; Additional File 1: Table S1). A closer look at the functional annotation of 23 protein-coding genes showed a candidate gene of interest, *Glyma.02G243200*, which encodes a protein with 64% homology to Arabidopsis MALE MEIOCYTE DEATH 1 (MMD1) protein. AtMMD1 is involved in the chromosomal condensation process during the microsporogenesis, and mutations in *MMD1* leads to male sterility in Arabidopsis [16–18].

### Identification of the *ms4* causal mutation by sequence comparison

**Fig. 3:**
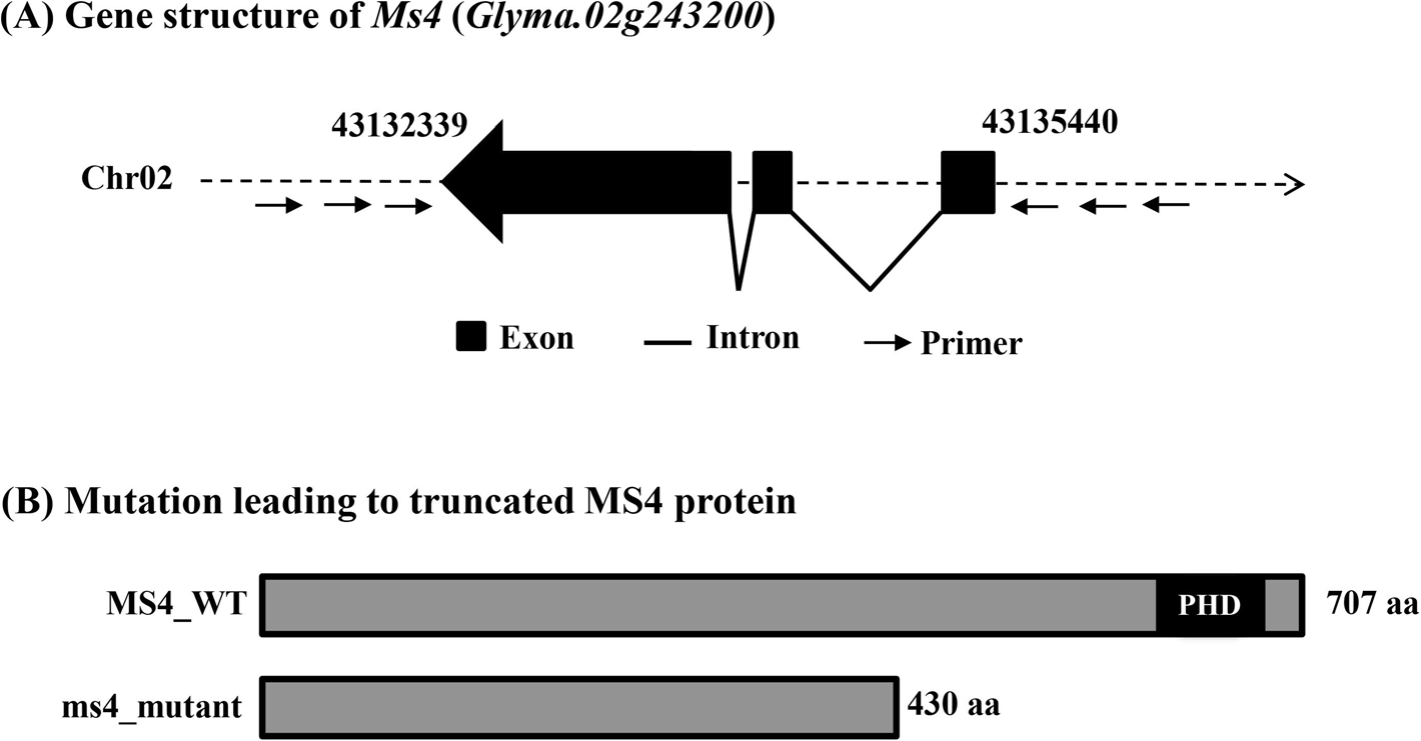
Gene and protein structure of the *Ms4* (*Glyma.02g243200*) locus. (**a**) *Glyma.02g243200* is 3.102 kb in length with 3 exons interrupted by 2 introns. (**b**) Comparison of the MS4 wild type (MS_WT) protein with the ms4 mutant (ms4_mutant) protein. An insertion event in *ms4* resulted in a truncated protein (ms4*_*mutant) that lacks the PHD domain

The candidate gene of interest *Glyma.02G243200* was successfully amplified from the genomic DNA of male-fertile (*Ms4/Ms4*) as well as homozygous male-sterile (*ms4/ms4*) soybean plants. Out of 9 different combinations of forward and reverse primers designed from 1,500 bp up- and down-stream sequences flanking the *Glyma.02G243200* gene, only MS4_F2 and MS4_R1 primer pair was able to amplify the expected fragment of 4.554 kb comprising 3.1 kb *Glyma.02G243200* gene (Fig. 3a; Additional File 1: Table S2A). The coding sequence comparison showed presence of three exons and two introns (Fig. 3a). The amplified fragment was sequenced with 12 internal primer pairs to cover the entire 3.1 kb region of *Glyma.02G243200* (Additional File 1: Table S2B). The nucleotide sequence of *Glyma.02G243200* from the male-sterile plant was compared with the male-fertile plants. The sequence comparison of the genomic DNA sequences showed an insertion of a single adenine (A) nucleotide in the 3rd exon in the male-sterile (*ms4/ms4*) line (Additional File 1: Figure S1). This single base insertion caused a frameshift mutation that resulted in a premature stop codon thereby forming a truncated protein of 430 amino acids (Fig. 3b; Additional File 1: Figure S2). Further, *Glyma.02G243200* transcripts from the wild type fertile and the *ms4* sterile mutants were also sequenced to validate the presence of the mutation and to confirm the exon-intron boundaries (data not shown).

### Phylogenetic analysis showed highly conserved nature of the MS4 proteins

The bioinformatic analysis of the MS4 protein sequence showed that it is a 707 amino acid long protein (Fig. 3b) with a theoretical molecular weight of 79.65 KDa and theoretical pI of 5.97 [19]. Further, conserved domain analysis showed that MS4 protein is a plant homeodomain (PHD)-finger protein as it contains a PHD_MMD1_like domain (CD15556) (Fig. 3b). The PHD domain is present at the C-terminus between 623 and 667 amino acid residues. The domain architecture shown by the soybean MS4 protein is consistent with its Arabidopsis counterpart PHD finger protein MALE MEIOCYTE DEATH 1 (AtMMD1). However, conserved domain analysis of the MS4 protein derived from male-sterile soybean line showed the absence of PHD domain in the truncated MS4 protein (Fig. 3b).

**Fig. 4:**
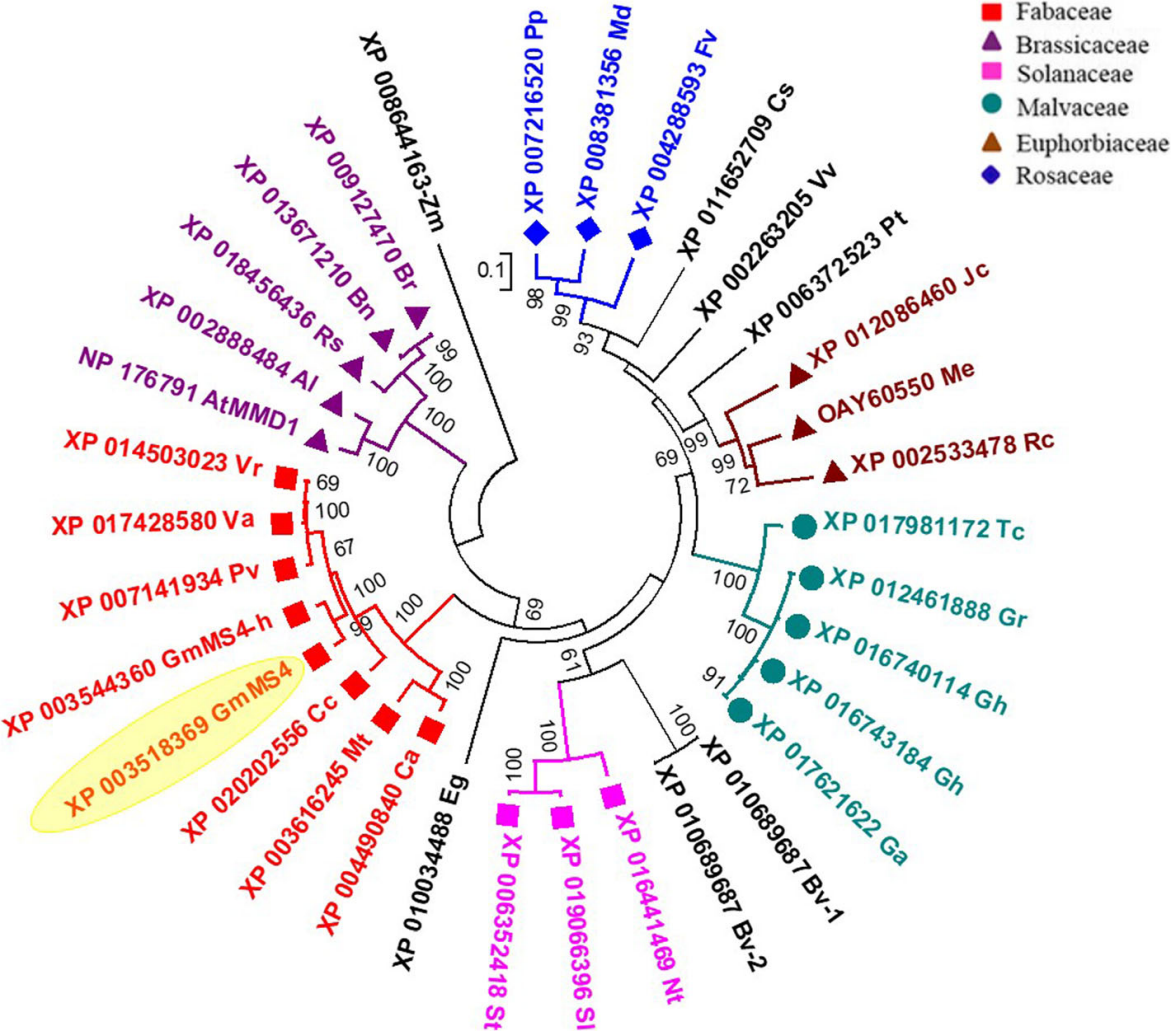
Phylogenetic tree of the soybean MS4 protein (Glyma.02g243200) and its homologs, identified using the BLAST analysis. The ClustalW aligned 34 protein sequences were used to construct the phylogenetic tree using the maximum likelihood method of MEGA v6.06. The soybean MS4 protein is highlighted in yellow. The abbreviations are: Al: *Arabidopsis lyrata*, At: *Arabidopsis thaliana*, Bv: *Beta vulgaris*, Bn: *Brassica napus*, Br: *Brassica rapa*, Cc: *Cajanus cajan*, Ca: *Cicer arientinum*, Cs: *Cucumis sativus*, Eg: *Eucalyptus grandis*, Fv: *Fragaria vesca*, Gm: *Glycine max*, Ga: *Gossypium arboreum*, Gh: *Gossypium hirsutum*, Gr: *Gossypium raimondii*, Jc: *Jatropha curcas*, Md: *Malus domestica*, Me: *Manihot esculentus*, Mt: *Medicago truncatula*, Nt: *Nicotiana tabacum*, Pv: *Phaseolus vulgaris*, Pt: *Populus trichocarpa*, Pp: *Prunus persica*, Rs: *Raphanus sativus*, Rc: *Ricinus communis*, Sl: *Solanum lycopersicum*, St: *Solanum tuberosum*, Tc: *Theobroma cacao*, Va: *Vigna angularis*, Vr: *Vigna radiata*, Vv: *Vitis vinifera*

The phylogenetic analysis was performed with the MS4 protein and its homologs identified in other plant species through protein basic local alignment search tool (BLAST) of NCBI. The phylogenetic tree showed clustering of the proteins based on their taxonomical relationship (Fig. 4). The soybean MS4 (XP_003518369) showed phylogenetic similarity with proteins derived from other legume species such as common bean (*Phaseolus vulgaris*; XP_007141934), mung bean (*Vigna radiata*; XP_014503023), adzuki bean (*V. angularis*; XP_017428580) and pigeon pea (*Cajanus cajan*; XP_020202556), etc. and all of them clustered into a Fabaceae specific cluster (Fig. 4). Surprisingly, we found another PHD domain containing protein (XP_003544360; MS4_homolog) in soybean with a high similarity with MS4 (Fig. 4). The MS4_homolog is a 708 aa long protein encoded by *Glyma.14G212300* (Gene: 3178 bp and CDS: 2127 bp) and had a sequence similarity of 95 and 92% with *Ms4’s* CDS and protein respectively (Additional File 1: Figure S3 and S4). Except MS4_homolog, the next closest protein was only 34.2% similar to MS4 protein which clearly shows MS4_homolog as the only homolog of MS4 in soybean (Data not shown). Further, both the genes were also present as duplicated gene pair in the same segmentally duplicated chromosomal block 245 (chr02-chr14) obtained from Plant Genome Duplication Database (Additional File 1: Table S3) which further confirms the similarity observed in phylogenetic analysis.

### *Ms4* is highly expressed during early flower development

**Fig. 5:**
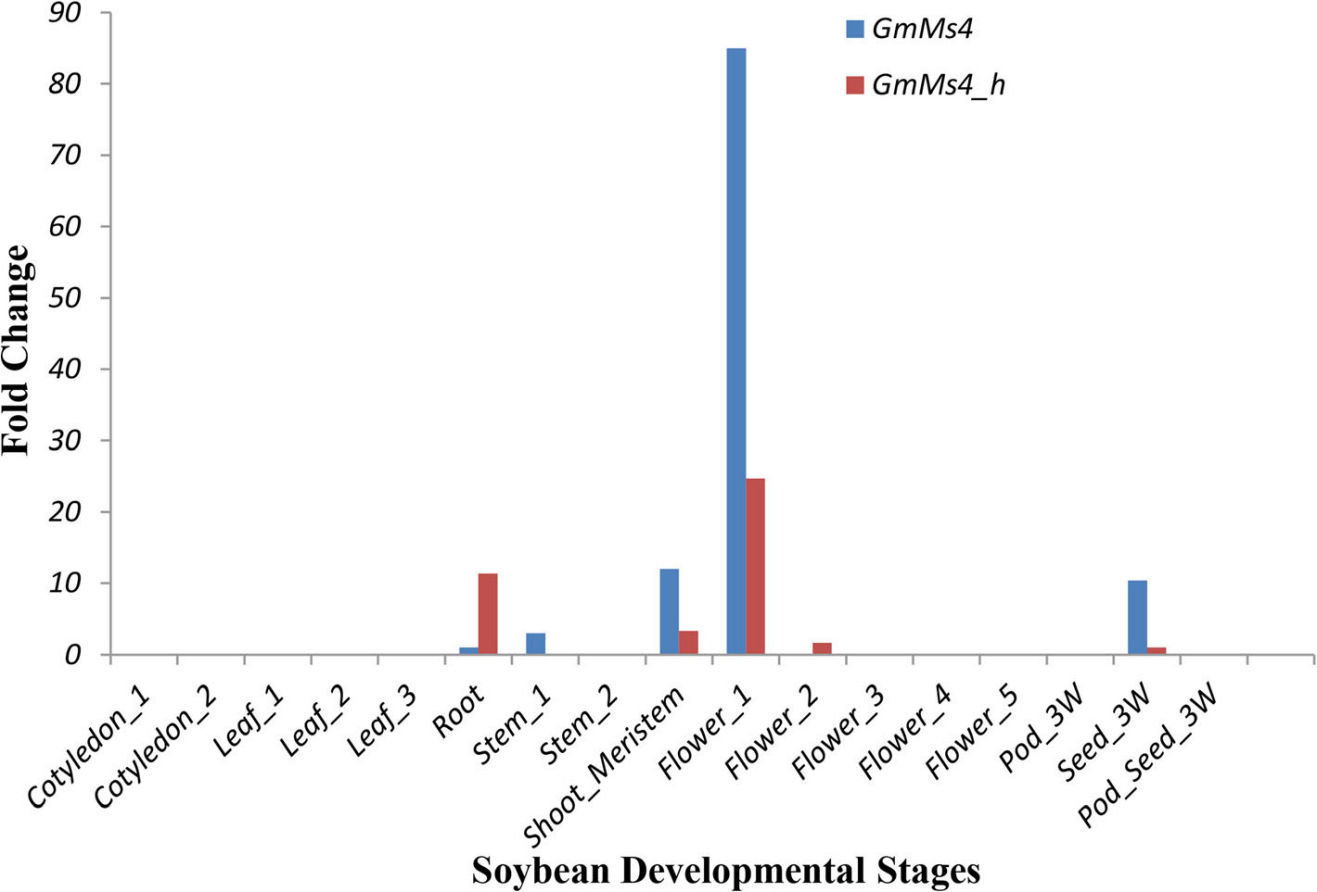
*Ms4* and *Ms4_h* expression across the various vegetative and reproductive stages of soybean development. Tissue with minimal expression of respective genes (root for *Ms4* and seed_3W for *Ms4_h)* were used as control to calculate fold change. Cotyledon_1: germination stage; cotyledon_2: trefoil stage; leaf_1: trefoil stage; leaf_2: flower bud differentiation stage; leaf_3: senescent leaves; root: germination stage; stem_1: germination; stem_2: trefoil stage; shoot_meristem: flower bud differentiation stage; flower_1: flower bud differentiation stage; flower_2: flowering stage-bud before flowering; flower_3: flowering stage-florescence; flower_4: flowering stage-5 d after flowering; flower_5: flowering stage-florescence-different stage; pod_3W: 3 weeks old pod; pod_seed_3W: 3 weeks old pod_seed; seed_3W: 3 weeks old seed

Since the PHD finger proteins are known to be involved in various functions in plants [20], we looked at the expression of *Ms4* and *Ms4_homolog* across various stages of plant development (Fig. 5). For this purpose, we used publicly available soybean RNA-seq datasets [21, 22]. The comparative expression analysis normalized to root tissue showed significantly higher expression of *Ms4* in early flowering (flower bud differentiation) stages followed by shoot meristematic tissue, 3 weeks old seed, stem and least expressed in root (Fig. 5). Similarly, comparative expression of *Ms4_homolog* normalized to seed_3w (lowest expression) also showed highest expression in early flowering (flower bud differentiation) stage however, significantly lower than that of *Ms4* (Fig. 5). Among the other tissues, *Ms4_homolog* was also expressed in roots, flower_2 with lowest in 3 weeks old seeds. Among the other 22 genes present in the mapped region, 21 were observed to express moderately across different stages whereas *Glyma.02G242500* was constitutively expressed across all the stages analyzed (Additional File 1: Figure S5).

### Functional characterization of *Ms4* using Arabidopsis model system

Soybean MS4 (*Glyma.02G243200*) and Arabidopsis MMD1 proteins shared 64% amino acid sequence similarity along with similar conserved domain structure. The Arabidopsis *MMD1* is an essential gene with role in male meiosis and homozygous *mmd1/mmd1* plants are male sterile [16]. Hence, we used the heterozygous Arabidopsis *mmd1* mutant for the complementation with the soybean genes for the functional characterization. *AtMMD1* native promoter driven genomic (Pro_*MMD1*_*::gMs4/ mmd1* and Pro_*MMD1*_*::gMs4_homolog*/ *mmd1*) and CDS (Pro_*MMD1*_*::cMs4/ mmd1* and Pro_*MMD1*_*::cMs4_homolog/ mmd1*) constructs were used to generate the transgenic plants (Additional File 1: Figure S6). Hygromycin selected T_1_ transgenic plants were also genotyped for the homozygous/ heterozygous *mmd1* mutant background. Three independent T_1_ transgenic lines for each construct were advanced to T_2_ generation to select the homozygous *mmd1/mmd1* plants and studied for complemented phenotype and microscopic analyses.

**Fig. 6:**
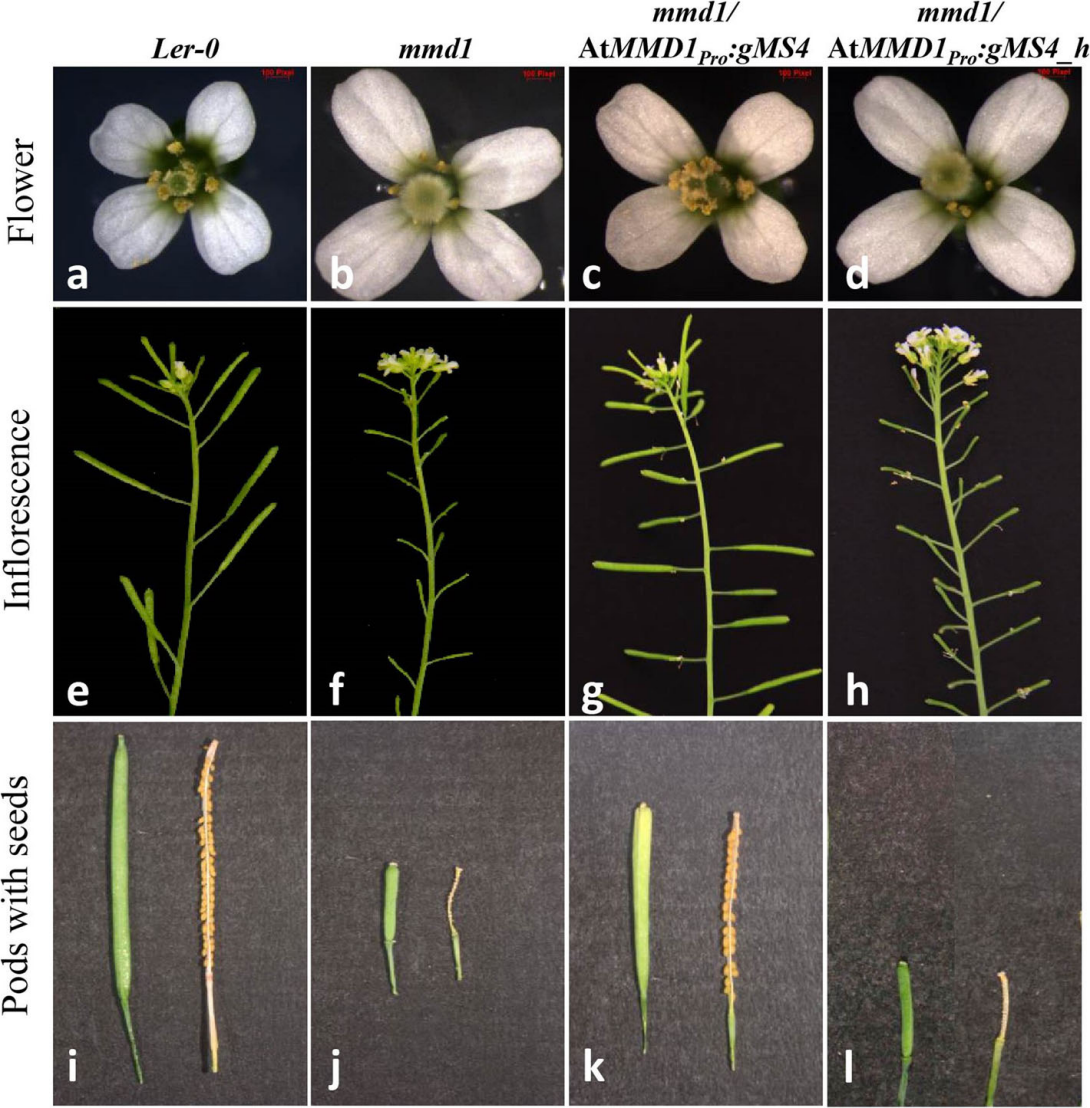
Comparative images of wild type (*Ler-0*), homozygous *mmd1* mutant, *mmd1* transformed with *Ms4* (*mmd1/*At*MMD1*_*Pro*_*:gMs4)* and *mmd1* transformed with *Ms4_homolog* (*mmd1/*At*MMD1*_*Pro*_*:gMs4_h)*. (**a-d**) Flowers with normal stamens (*Ler-0* and *Ms4* transformed *mmd1* plants) and defective stamens (*mmd1* mutant and *Ms4_homolog* transformed *mmd1* plants). (**e-h**) Fertile Arabidopsis plants with successful pod formation (*Ler-0* and *Ms4* transformed *mmd1* plants) and sterile plants with no pod formation (*mmd1* mutant and *Ms4_homolog* transformed *mmd1* plants). (**i-l**) Pods with seeds (*Ler-0* and *Ms4* transformed *mmd1* plants) and pods without seeds (*mmd1* mutant and *Ms4_homolog* transformed *mmd1* plants). For Fig. 6 l, we have combined two different pictures to bring uniformity

**Fig. 7:**
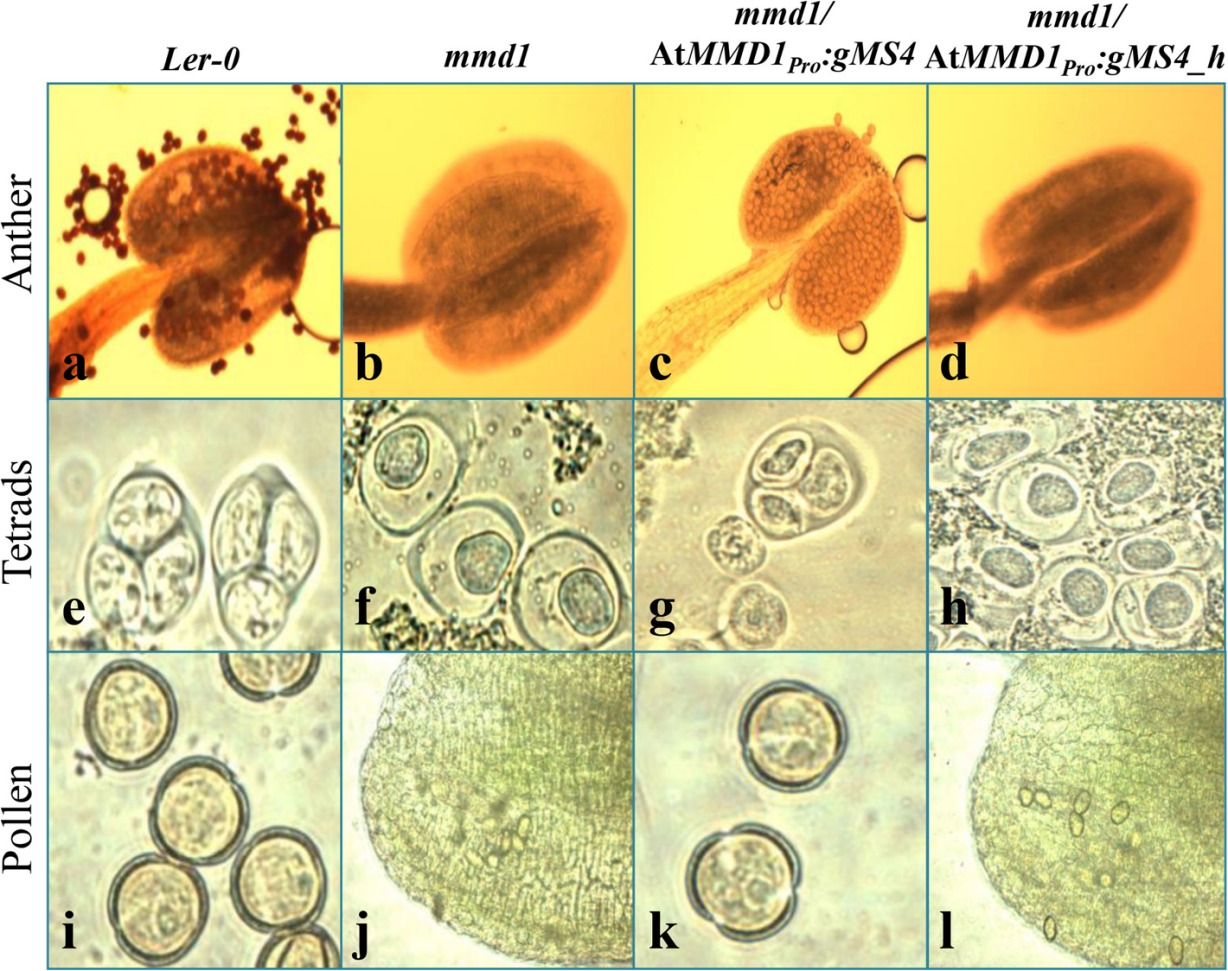
Comparative microscopic images of different stages of pollen development from Arabidopsis wild type (*Ler-0*), homozygous *mmd1* mutant and *mmd1* transformed with *Ms4* (*mmd1/*At*MMD1*_*Pro*_*:gMs4)* and *mmd1* transformed with *Ms4_homolog* (*mmd1/*At*MMD1*_*Pro*_*:gMs4_h)*. (**a-d**) Anthers stained with I_2_KI. (**e-h**) Tetrad formation. (**i-l**) Pollen

The homozygous *mmd1/mmd1* plants carrying the *Ms4* constructs (Pro_*MMD1*_*::gMs4* and Pro_*MMD1*_*::cMs4*) showed successful complementation of male-sterile phenotype of *mmd1* mutant, whereas the homozygous *mmd1/mmd1* plants carrying *Ms4_homolog* constructs (Pro_*MMD1*_*::gMs4_homolog* and Pro_*MMD1*_*::cMs4_homolog*) did not complement the male-sterile phenotype (Fig. 6; Data not shown for the cDNA complementation). Further, the *Ms4* complemented plants produced siliques with viable seeds (Fig. 6 e, g, i and k) whereas the siliques of *Ms4_homolog* complemented plants were similar to the *mmd1* mutant (Fig. 6 f, h, j and l). Siliques of the *Ms4* complemented plants were observed slightly shorter in size as compared to the *Ler-0* plants (Fig. 6 i and k). Since the *mmd1/mmd1* mutant produced shorter filaments and pollen-less stamens placing the anthers below the stigma, we further analyzed the flowers from the *Ms4* and *Ms4_homolog* complemented lines for the differences in the stamens. The microscopic images showed that the flowers of the *Ms4* complemented lines produced normal stamens with normal filaments and viable pollens similar to control (*Ler-0*) whereas the flowers of the *Ms4*_*homolog* complemented lines were shorter and pollen-less stamens similar to the homozygous *mmd1* mutants (Fig. 6 a-d and Fig. 7 a-d). To further confirm the complementation of homozygous *mmd1* defective microspore development, we performed the comparative microscopic analysis of the anther developmental stages of the complemented lines with the Arabidopsis *Ler-0* and homozygous *mmd1* mutant. The *Ms4* complemented *mmd1* plants showed microspores with successful tetrad formation which were released successfully from anthers whereas the *Ms4_homolog* complemented *mmd1* plants showed microspores with shrunken cytoplasm at stage 4 of the anther development, which degenerated and failed to release at stage 8 as observed in anthers from *mmd1* homozygous mutant (Fig. 7 e-l). However, there were fewer tetrads and fewer pollens observed in the *Ms4* complemented plants compared to the wild type plants (Fig. 7 e-l).

## Discussion

Hybrid seed production and utilization has revolutionized the crop production by exploiting the genetic phenomenon of heterosis/hybrid vigor. This technological advancement directly contributed to an increase in the yield as seen in rice where hybrid rice yield increased ~ 10–20% over the conventional lines [23]. There is a high potential to exploit the heterosis in soybean; hence, understanding molecular basis of male sterility in soybean mutant lines is critical for the development of high yielding hybrid varieties. Further, insects have shown to be involved in transferring pollen from male-fertile plants to male-sterile soybean plants [24] which avoids laborious manual pollination. Identification and characterization of soybean male-sterile lines are critical for producing hybrid seed at commercial level as well as to understand the mechanism responsible for male sterility. In addition, the genetic male sterility has an advantage over the cytoplasmic male sterility (CMS) that it does not require a dedicated maintainer line to propagate the male-sterile line. The major constraint in the utilization of the genetic male sterility for production of pure hybrid seeds is the identification and removal of the male-fertile plants before flowering. Successful pre-flowering recognition of homozygous male sterile plants requires specific morphological or molecular markers. Male-sterile lines often do not display visible phenotype before flowering hence the gene-specific molecular markers are more practical in the identification and removal of male-fertile plants. In addition, these markers can be used for male-sterile line selection in backcrossing and recurrent selection breeding programs [3]. Further, identification of photoperiod/thermo-sensitive genic male sterile (PTGMS) lines may provide flexibility in regulating fertility by managing environmental conditions [25]. Hence, developing independent male-sterile lines and understanding the molecular mechanisms will help to deploying a suitable approach for hybrid seed production. In soybean, eleven independent male-sterile lines (*ms1, ms2, ms3, ms4, ms5, ms6, ms7, ms8, ms9, msMOS,* and *msp*) generated by various methods (spontaneous, fast neutron irradiation and transposable elements) have been identified [6–8]. In addition, the availability of the male-sterile lines from diverse genetic backgrounds further facilitates the opportunity for the exploitation of genetic diversity from different lines. Despite the identification of several male-sterile lines in soybean, not much is known about the identity of the genes or molecular mechanism behind the male sterility.

The overarching goal of the present study was to identify and characterize the gene responsible for male-sterile phenotype of the *ms4* mutant. The *ms4* was previously mapped to a 694 kb region on chromosome 2 which harbored 88 genes [6]. The soybean *msp* gene was also mapped in the same region; hence fine mapping to identify the actual causal gene was essential to develop gene-specific molecular markers [6]. It is believed that *ms4* is different from *msp,* as the mechanisms involved in governing male sterility are different [6] indicating that mutations in independent loci are responsible for the male sterility in these lines. The *msp* is a temperature sensitive male sterile mutant, which displays higher fertility in the hot environment compared to cooler temperatures [14], while *ms4* is not a temperature sensitive mutant. In the present study, we have fine mapped the *ms4* locus to ~ 216 kb region that contained 23 protein-coding genes (Fig. 2; Additional File 1: Table S1) and candidate gene was identified based on the functional relevance. The candidate gene (*Glyma.02G243200)* displayed high homology to Arabidopsis *MMD1* which is involved in the male fertility in Arabidopsis [16–18]. The PHD domain protein AtMMD1 has been shown to be essential for proper chromosome condensation during male meiosis [16–18]. The *mmd1* mutant fails to produce viable pollen due to pollen degeneration after the tetrad stage, a phenomenon that is also observed in the *ms4* mutants, endorsing *Glyma.02G243200* as the candidate gene. Sequence analysis of *Glyma.02G243200* from *ms4/ms4* plants confirmed the presence of a spontaneous mutation resulting into a truncated protein lacking PHD domain (Fig. 3B; Additional File 1: Figure S1 and S2) [17, 18]. The PHD domain has been shown to be critical for MMD1 function in Arabidopsis [16, 18] which is missing in *ms4/ms4* plants due to spontaneous mutation resulting in truncated protein.

Interestingly, phylogenetic analysis of the MS4 protein revealed the presence of another PHD domain protein, MS4_homolog, encoded by *Glyma.14G212300* gene on chromosome 14. These two homologs appear to be result of segmental duplication during paleoploidization event (Additional File 1: Table S3). Even though cytogenetically soybean behaves as diploid, there are reports of at least two rounds of whole genome duplication [26, 27]. It is interesting that despite the presence of a homolog, the *Ms4* is the only gene that is governing the male-sterile phenotype. These results are supported by the expression data which shows that soybean *Ms4* expression is significantly higher than *Ms4_homolog* in most of the tissues, except root (Fig. 5). There are several reports of similar expressional shift (spatial and quantitative) among the remained duplicated genes (paralogs) in plants including soybean [28]. Non-functionality of *MS4_homolog* can also be explained based on the study in which loss or silencing of approximately 25% of the duplicated genes in soybean were reported since the last duplication event [26, 28]. The Arabidopsis homozygous mutant lines (*mmd1/mmd1*) complemented with soybean *Ms4* (*AtMMD1*_*pro*_*::gMs4* and *AtMMD1*_*pro*_*::cMs4*) showed successful functional complementation by producing viable pollen and siliques with seeds, whereas the *Ms4_homolog* was not able to complement (Fig. 6). Since, both *Ms4* and *Ms4_homolog* genes were driven by native *MMD1* promoter, the functional characterization clearly showed lack of function for *Ms4_homolog*, which explains the reason behind the male sterile phenotype of *ms4* despite the presence of another homolog. The failure of *Ms4_homolog* to rescue the male-fertile phenotype could be attributed to the 7% differences in amino acid composition between these two proteins (Additional File 1: Figure. S4). Further studies are needed to understand the reasons for the functional differences. In addition to its inability to produce viable pollens, homozygous *mmd1* mutants produce shorter filaments placing the anthers below the stigma (Fig. 6). The complemented *Ms4* lines produced normal filaments and viable pollens suggesting that the PHD finger proteins are involved in the filament elongation in addition to the chromosomal condensation (Fig. 6). Overall, our data successfully demonstrated that the spontaneous mutation in *Glyma.02G243200* that resulted in premature stop codon is responsible for the male-sterile phenotype of the soybean *ms4* line.

## Conclusions

Stable male sterility is an important component of an effective hybrid system in self-pollinated crops. This is the first report on map based cloning and characterization of a soybean gene responsible for male fertility. Successful utilization of the male sterility phenomenon requires a complete understanding of associated mechanisms in soybean, which warrants cloning, and characterization of the existing male-sterile lines apart from generating new male-sterile lines. The current study revealed that a similar molecular mechanism is involved in determining the male-sterile phenotype in soybean and Arabidopsis. Information from other plant species can also be utilized to make rapid progress in understanding the male sterility mechanism in soybean through translational research. Employing the model system will result in rapid progress in understanding the molecular mechanism involved in male-sterile phenotypes. A comprehensive understanding of the molecular mechanisms and identification of genes responsible for the male-sterile phenotypes will help to utilize the best system for the production of efficient and pure hybrid seeds in soybean.

## Methods

### Development of mapping population and molecular mapping

The mapping population was generated by crossing T274H (*ms4/ms4*) as a female parent with Manchu (*Ms4/Ms4*) as a male parent. The parental lines were obtained from the USDA Soybean Germplasm Collection [29]. The resulting F_1_ plants were self-pollinated to generate F_2_ mapping population, which was used for molecular mapping experiment. Fertile/sterile nature of the segregating population was tested by staining the pollen with potassium iodine solution [30] and also by observing the seed set at the harvest. Upon staining, the fertile pollen turns into dark red-brown while the sterile pollen appears light in color and translucent. The *ms4/ms4* plants did not produce the pods; however, *Ms4/ms4* and *Ms4/Ms4* plants produced the pods with seeds (Fig. 1).

Genomic DNA was isolated from the F_2_ plants using CTAB method as described earlier [31]. The sequence information of SSR (Simple Sequence Repeat) markers was obtained from Soybase database [32, 33]. For microsatellite analysis using PCR, 30 ng genomic DNA was used as template in a 10 μl PCR reaction containing 1x reaction buffer (10 mM Tris-HCl, 50 mM KCl, pH 8.3), 2.0 mM MgCl_2_; 0.25 μM of each primer; 200 μM of each dNTP and 0.25 units of *Biolase* DNA polymerase (Bioline, USA Inc., Taunton, MA). The PCR conditions used were as follows: 2 min at 94 °C; 35 cycles of 30 s at 94 °C, 30 s at 58 °C and 1 min at 72 °C, followed by 8 min at 72 °C. The amplified PCR products were separated on a 4% agarose gel. The Mapmaker 2.0 program was used to determine genetic linkages and genetic distances [34]. Marker order was determined at a LOD threshold of 3.0. Linkage calculations were done using the Kosambi mapping function [35].

### Amplification and characterization of the candidate *ms4* gene (*Glyma.02G243200*)

Nine combinations of forward and reverse primers were used to amplify the gene from the male-fertile (*Ms4/Ms4*) and the male-sterile (*ms4/ms4*) plants (Additional File 1: Table S2A). Long-range PCR was performed using touchdown PCR method with an annealing temperature ranging from 68 °C to 60 °C and extension temperature of 72 °C. PCR products were separated on a 0.8% agarose gel, fragments were excised and purified using gel purification kit (IBI Scientific, Peosta, IA, USA). Twelve pairs of internal primers were used to sequence the full-length *Ms4* gene (Additional File 1: Table S2B). The amplified PCR products were sequenced using Sanger sequencing at Functional Biosciences (Madison, WI) and assembled using Vector NTI program (Life Technologies Corporation, Grand Island, NY, USA). *Ms4* genomic DNA and cDNA sequences derived from wild type soybean were compared to each other to identify the exon-intron boundaries using Exon-Intron Graphic Maker [36]. Assembled *Ms4* genomic DNA sequences from fertile and sterile soybean plants were compared to determine the causal mutation (insertion). Further, the full length and the truncated MS4 protein sequences from the fertile and the sterile soybean lines were aligned using ClustalW, respectively and conserved domains were identified using NCBI Conserved Domains Database (CDD) [37]. Additionally, the physical properties, like molecular weight and pI value of the MS4 protein were also analyzed using ExPASy Compute pI/Mw tool [38].

### Phylogenetic analysis

The amino acid sequence of the MS4 protein was used to identify the homologs in soybean as well as other plant species using BlastP search against the public domain database [39]. The identified 33 MS4 related protein sequences along with the soybean MS4 sequence were aligned using MEGA inbuilt ClustalW alignment tool with standard parameters. Further, the aligned sequences were used to build the maximum likelihood phylogenetic tree using MEGA v6.06 software [40] with 1000 times boot strap replications. Other parameters used for tree construction were: Substitution type: Amino acid; Model: Equal input model; Rates among sites: Uniform rates; Gaps/missing data treatment: Partial deletion; Site coverage cutoff: 95%.

### Developmental stage specific expression analysis

To perform the soybean *Ms4* and *Ms4_h* expression analysis, 17 publicly available RNA-seq datasets related to various vegetative (cotyledon (2), leaf (3), stem (2), root and shoot_meristem) and reproductive (flower (5), pod, seed and pod_seed) stages of soybean development were downloaded from the NCBI SRA database (accession PRJNA238493) (Additional file 1: Table S4). All the datasets were analyzed using RNA seq analysis tool of CLC Genomics workbench. Expression data related to *Ms4* and *Ms4_homolog* was extracted, and fold change was calculated using the tissue with minimum expression as control. A heat map was plotted for the expression data of 22 other genes present in the mapped region using the online tool Morpheus (https://software.broadinstitute.org/morpheus/).

### Complementation vector construction

Soybean plants (Dyna-Gro S56RY84) were grown under controlled greenhouse conditions at 28 °C temperature. Leaves and flower buds were collected from the flowering stage plants and flash frozen using liquid nitrogen and stored at − 80 °C. The genomic DNA from leaf tissue was extracted using DNeasy kit (Qiagen, Valencia, CA USA). RNA from flower buds was extracted using the plant spectrum total RNA isolation kit (Sigma Aldrich) and simultaneously treated with on column DNaseI (Sigma Aldrich) using standard protocol. Further, 1 μg of DNaseI treated RNA was used for the cDNA synthesis using the SuperScript II reverse transcriptase (Invitrogen). The *Ms4* (*Glyma.02G243200*) and its homologous gene (*Ms4_homolog; Glyma.14G212300*) sequences obtained from phytozome soybean genome sequence data (https://phytozome.jgi.doe.gov/pz/portal.html) were used to design primers to amplify the full-length sequence for cloning and complementation studies (Additional File 1: Table S2C). Since both the genes share very high sequence similarity at the 5′ and 3′ ends, same primer pair was used to amplify both the genes. To drive the expression of both the genes, Arabidopsis native *MMD1* promoter was amplified using the specific primer pair (Additional File 1: Table S2C) and cloned into pMDC32 expression vector using *Sbf*I and *Asc*I restriction enzymes (New England BioLabs, Ipswich, MA, USA). The *Ms4* and *Ms4_homolog* genes (genomic DNA fragments) were amplified using NEBNext® High-Fidelity 2x PCR Master Mix (New England BioLabs, Ipswich, MA, USA) with the PCR condition as follows: 3 min at 98 °C; 30 cycles of 30 s at 98 °C, 30 s at 58 °C and 2 min at 72 °C, followed by 10 min at 72 °C. The amplified products were separated on a 1% agarose gel and DNA fragments of ~ 3.1 kb (gDNA) and ~ 2.1 kb (cDNA) were gel purified using a GeneJET Gel extraction kit (Thermo Fisher Scientific, Carlsbad, CA, USA). The purified PCR products were cloned into the expression vector pMDC32 containing Arabidopsis *MMD1* native promoter using *Asc*I and *Pac*I restriction enzymes and T4 DNA ligase (NEB, Ipswich, MA, USA). The ligation mix was transformed into DH5-α chemical competent *Escherichia coli* cells. Plasmid was extracted from the colony PCR positive colonies using GeneJET Plasmid Miniprep kit (Thermo Fisher Scientific, Vilnius, Lithuania) and further confirmed by restriction digestion with *Asc*I and *Pac*I enzymes. The *Ms4* and *Ms4_homolog* specific constructs were confirmed using the restriction digestion with *Hin*dIII which is only present in the *Ms4* and not in *Ms4_homolog*. The sequencing confirmed genomic (*AtMMD1:gMs4* and *AtMMD1:gMs4_homolog*) and CDS (*AtMMD1:cMs4* and *AtMMD1:cMs4_homolog*) constructs were transformed into *Agrobacterium tumefaciens* using standard transformation protocol and PCR positive colonies were used for plant transformations.

### Plant transformation

Complementation studies were performed using the *male meiocyte death 1* (*mmd1*) mutant of *Arabidopsis thaliana* ecotype *Landsberg erecta* (*Ler-0*) [16]. Since, the homozygous *mmd1* is male sterile, only heterozygous (*MMD1/mmd1*) plants can be transformed. The mutant plants were genotyped for heterozygous nature before transformation using gene-specific forward and dissociator (Ds) specific reverse primer pairs as described previously [16]. The genotyped *mmd1* heterozygous plants were transformed with soybean *Ms4* and *Ms4_homolog* genomic (*AtMMD1:gMs4* and *AtMMD1:gMs4*_*homolog*) and CDS (*AtMMD1:cMs4* and *AtMMD1:cMs4_homolog*) complementation constructs using *A. tumefaciens* (GV3001) mediated floral dip transformation method [41]. The T_1_ transgenic seeds were grown on Murashige and Skoog (MS) media with Hygromycin (50 μg/ml) to select the transformants. The selected T_1_ transgenic plants were advanced to T_2_ generation to isolate homozygous lines which were used for further functional characterization.

### Microscopy analysis

Mature flower and silique samples were collected from the *Ler-0*, homozygous mutant (*mmd1/mmd1*), *Ms4* (*mmd1/AtMMD1:gMs4* and *mmd1/AtMMD1:cMs4*) and *Ms4_homolog* (*mmd1/AtMMD1:gMs4_homolog* and *mmd1/AtMMD1:cMs4_homolog*) homozygous transgenic lines to image stamens, pollens using light microscope BX-41 (Olympus, MA, USA) and siliques using SZ61 stereomicroscope (Olympus, MA, USA). Anthers were separated from the flower and stained with I_2_KI solution [42]. Dark red color staining, an indicator of live pollen was used to compare the pollen from *Ler-0*, mutant and complemented lines. Microspore development was studied at stage 4 and stage 8 of the anther development, using light microscopy [43].

## Additional file

Additional file 1: **Figure S1.** Sequence comparison of the genomic DNA sequences showed an insertion of a single nucleotide “A” in the 3rd exon of the *Ms4* gene (*Glyma.02G243200)* in the male-sterile (*ms4/ms4*) line. Green color boxes represent exonic sequences. Insertion mutation is shown by black color box. **Figure S2.** Amino acid alignment showing a frameshift mutation resulted in an early stop codon in *ms4* sterile mutant. The resulting truncated protein lacks the Plant Homeodomain (PHD) which is otherwise present at the C-terminus of native MS4 protein. **Figure S3.** Sequence comparison of the soybean *Ms4* and *Ms4_homolog* coding sequences. **Figure S4.** Similarity comparison of the soybean MS4 and MS4_homolog amino acid sequences. **Figure S5.** Heatmap representing the expression of the remaining 22 genes present in the mapped region. **Figure S6.** Constructs used for the complementation purposes. (A) Arabidopsis MMD1 native promoter driven genomic and CDS *Ms4* constructs. (B) Arabidopsis MMD1 native promoter driven genomic and CDS *Ms4_homolog* constructs. **Table S1.** A list of 23 predicted genes in the *ms4* region flanked by BARCSOYSSR_02_1515 and BARCSOYSSR_02_ 1528. **Table S2.** Details of primers used in present study for various purposes. **Table S3.** Duplication gene list of syntenic block 245 between soybean chr02-chr14 with *Ms4* and *Ms4_homolog* as duplicated gene pair (highlighted in yellow). **Table S4.** List of SRA files used for the expression analysis of *Ms4* and *Ms4_h*. (PDF 672 kb)

## Data Availability

We have used publicly available RNA-seq data from the NCBI SRA database BioProject: PRJNA238493. More details are available in Additional file 1: Table S4.

## Abbreviations

BLAST: Basic local alignment search tool
CDS: Coding sequence
MMD1: MALE MEIOCYTE DEATH 1
MS: Male sterile
PHD: Plant Homeodomain
