# Supplementary material for: Mutation in a PHD-finger protein MS4 causes male sterility in soybean
Source: BMC Plant Biol. 2019 Aug 28;19:378. doi: 10.1186/s12870-019-1979-4 (PMC6712664; doi:10.1186/s12870-019-1979-4)
Supplement: Supplementary file 1 — Figure S1. Sequence comparison of the genomic DNA sequences showed an insertion of a single nucleotide “A” in the 3rd exon of the Ms4 gene (Glyma.02G243200) in the male-sterile (ms4/ms4) line. Green color boxes represent exonic sequences. Insertion mutation is shown by black color box. Figure S2. Amino acid alignment showing a frameshift mutation resulted in an early stop codon in ms4 sterile mutant. The resulting truncated protein lacks the Plant Homeodomain (PHD) which is otherwise present at the C-terminus of native MS4 protein. Figure S3. Sequence comparison of the soybean Ms4 and Ms4_homolog coding sequences. Figure S4. Similarity comparison of the soybean MS4 and MS4_homolog amino acid sequences. Figure S5. Heatmap representing the expression of the remaining 22 genes present in the mapped region. Figure S6. Constructs used for the complementation purposes. (A) Arabidopsis MMD1 native promoter driven genomic and CDS Ms4 constructs. (B) Arabidopsis MMD1 native promoter driven genomic and CDS Ms4_homolog constructs. Table S1. A list of 23 predicted genes in the ms4 region flanked by BARCSOYSSR_02_1515 and BARCSOYSSR_02_ 1528. Table S2. Details of primers used in present study for various purposes. Table S3. Duplication gene list of syntenic block 245 between soybean chr02-chr14 with Ms4 and Ms4_homolog as duplicated gene pair (highlighted in yellow). Table S4. List of SRA files used for the expression analysis of Ms4 and Ms4_h. (PDF 672 kb) [file 12870_2019_1979_MOESM1_ESM.pdf]

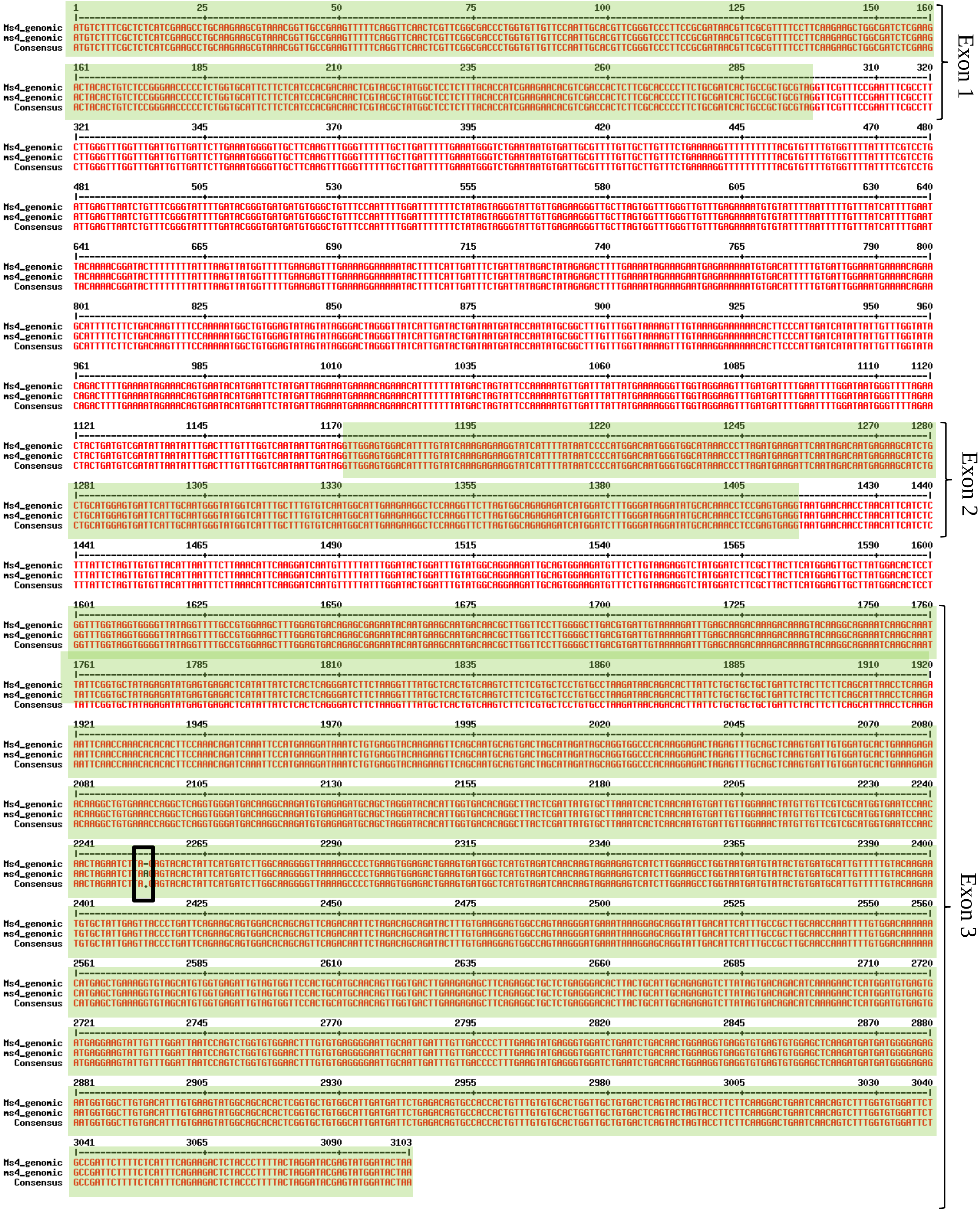

Figure S1. Sequence comparison of the genomic DNA sequences showed an insertion of a single nucleotide "A" in the 3rd exon of the *MS4* gene *Glyma.02G243200* in the male sterile (*ms4/ms4*) line. Green color boxes represent exonic sequences. Insertion mutation is shown by black color box.

```
Wild type (MS4) MSFALIEACKKRKRLPKFFRFNSFGDPGVVPIARSGPFRDNVRVFLQEAGDLEDYTVSGN
Mutant    (ms4) MSFALIEACKKRKRLPKFFRFNSFGDPGVVPIARSGPFRDNVRVFLQEAGDLEDYTVSGN
                ************************************************************

Wild type (MS4) PLWCILLIHDNSYAMAPLYTIEEHVDHSSHPFCDHCRCVGWSGHFVSKRRYHFIIPMDNG
Mutant    (ms4) PLWCILLIHDNSYAMAPLYTIEEHVDHSSHPFCDHCRCVGWSGHFVSKRRYHFIIPMDNG
                ************************************************************

Wild type (MS4) WHKPLDEDSIDNEKHLLHGVIHCNGYGHLLCVNGIEEGSKVLSGREIMDLWDRICTNLRV
Mutant    (ms4) WHKPLDEDSIDNEKHLLHGVIHCNGYGHLLCVNGIEEGSKVLSGREIMDLWDRICTNLRV
                ************************************************************

Wild type (MS4) RKIAVEDVSCKRSMDLRLLHGVAYGHSWFGRWGYRFCRGSFGVTEREYNEAMTTLGSLGL
Mutant    (ms4) RKIAVEDVSCKRSMDLRLLHGVAYGHSWFGRWGYRFCRGSFGVTEREYNEAMTTLGSLGL
                ************************************************************

Wild type (MS4) DVIVKDLSKTKTKYKAEIKQIIRCYRDMSETHIISLRDLLRFMLTVKSSRAPVPKITDTY
Mutant    (ms4) DVIVKDLSKTKTKYKAEIKQIIRCYRDMSETHIISLRDLLRFMLTVKSSRAPVPKITDTY
                ************************************************************

Wild type (MS4) SAAADSTSSALTSRNSTKHTLPNRSNSMKDKSVRYKKFSNAVTSIDSRWPTRRLEFAAQV
Mutant    (ms4) SAAADSTSSALTSRNSTKHTLPNRSNSMKDKSVRYKKFSNAVTSIDSRWPTRRLEFAAQV
                ************************************************************

Wild type (MS4) IVDALKENKAVKPGSGGMTRQDVRDAARIHIGDTGLLDYVLKSLNNVIVGNYVVRRMVNP
Mutant    (ms4) IVDALKENKAVKPGSGGMTRQDVRDAARIHIGDTGLLDYVLKSLNNVIVGNYVVRRMVNP
                ************************************************************

Wild type (MS4) TTRILEYTIHDLGKGLKAPEVETEVMAHVDQQVEESSWKPGNDVYCDALFLYKNVLLSYP
Mutant    (ms4) TTRILRVHYS*--------------------------------------------------
                *****.

Wild type (MS4) DSEAVDTAVQTILDSRYFVKEWPVRDEIKEQVLTFICRLQPNFVDKKHELKGVACGEIVV
Mutant    (ms4) ------------------------------------------------------------

Wild type (MS4) VPLHATVGDLKRASEAALRDTYCIAESLIVTDIKELMDVSDEEVLFGLIQSGVELCVRGI
Mutant    (ms4) ------------------------------------------------------------

                                                Plant Homeodomain
Wild type (Ms4) AIDLLTPLKYEGGSESDNWKVRCECGAQDDDGERMVACDICEVWQHTRCCGIDDSETVPP
Mutant    (ms4) ------------------------------------------------------------

Wild type (MS4) LFVCTGCCDSVLVPSSRTESTVFGVDSADSFLISEDSTLLLGYEYGY
Mutant    (ms4) -----------------------------------------------
```

Figure S2. Amino acid alignment showing a frameshift mutation resulted in an early stop codon in *ms4* sterile mutant. The resulting truncated protein lacks the Plant Homeodomain (PHD) which is otherwise present at the C-terminus of native MS4 protein.

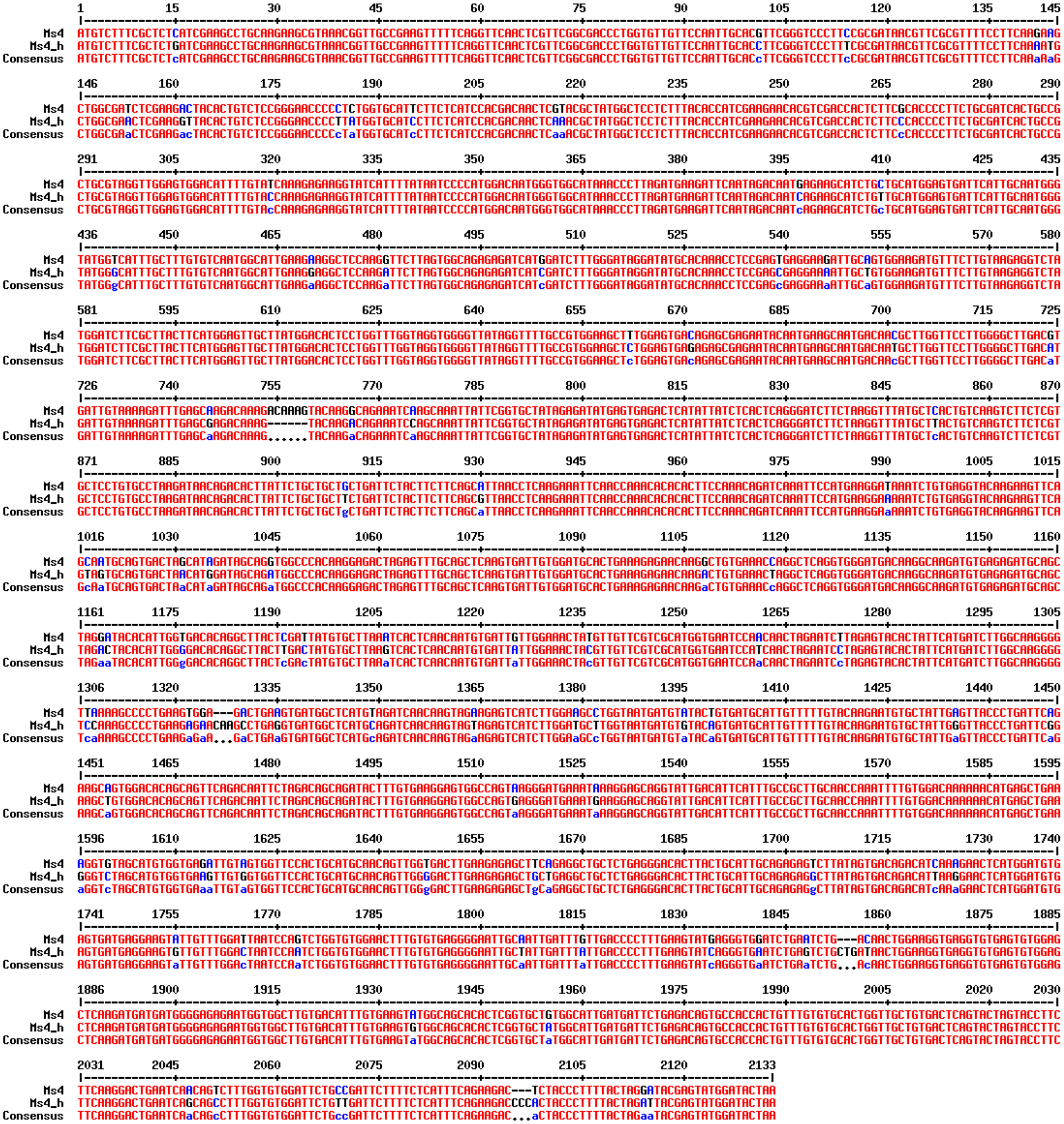

Figure S3. Sequence comparison of the soybean *MS4* and *MS4_homolog* coding sequences.

```
           1       10        20        30        40        50        60        70        80        90       100
           |--------+---------+---------+---------+---------+---------+---------+---------+---------+---------|
      MS4  MSFALIEACKKRKRLPKFFRFNSFGDPGVVPIARSGPFRDNVRVFLQEAGDLEDYTVSGNPLWCILLIHDNSYAMAPLYTIEEHVDHSSHPFCDHCRCVG
    MS4_h  MSFALIEACKKRKRLPKFFRFNSFGDPGVVPIAPSGPFRDNVRVFLQNAGELEGYTVSGNPLWCILLIHDNSNAMAPLYTIEEHVDHSSHPFCDHCRCVG
Consensus  MSFALIEACKKRKRLPKFFRFNSFGDPGVVPIArSGPFRDNVRVFLQ#AG#LEdYTVSGNPLWCILLIHDNSnAMAPLYTIEEHVDHSSHPFCDHCRCVG

           101    110       120       130       140       150       160       170       180       190       200
           |--------+---------+---------+---------+---------+---------+---------+---------+---------+---------|
      MS4  WSGHFVSKRRYHFIIPMDNGWHKPLDEDSIDNEKHLLHGVIHCNGYGHLLCVNGIEEGSKVLSGREIMDLWDRICTNLRVRKIAVEDVSCKRSMDLRLLH
    MS4_h  WSGHFVPKRRYHFIIPMDNGWHKPLDEDSIDNQKHLLHGVIHCNGYGHLLCVNGIEGGSKILSGREIIDLWDRICTNLRARKIAVEDVSCKRSMDLRLLH
Consensus  WSGHFVpKRRYHFIIPMDNGWHKPLDEDSIDN#KHLLHGVIHCNGYGHLLCVNGIEeGSK!LSGREIiDLWDRICTNLRaRKIAVEDVSCKRSMDLRLLH

           201    210       220       230       240       250       260       270       280       290       300
           |--------+---------+---------+---------+---------+---------+---------+---------+---------+---------|
      MS4  GVAYGHSWFGRWGYRFCRGSFGVTEREYNEAMTTLGSLGLDVIVKDLSKTKTKYKAEIKQIIRCYRDMSETHIISLRDLLRFMLTVKSSRAPVPKITDTY
    MS4_h  GVAYGHSWFGRWGYRFCRGSSGVREREYNEAMTMLGSLGLDMIVKDLSETK--YKTEIQQIIRCYRDMSETHIISLRDLLRFMLTVKSSRAPVPKITDTY
Consensus  GVAYGHSWFGRWGYRFCRGSfGVrEREYNEAMTmLGSLGLDmIVKDLSeTK..YKaEIqQIIRCYRDMSETHIISLRDLLRFMLTVKSSRAPVPKITDTY

           301    310       320       330       340       350       360       370       380       390       400
           |--------+---------+---------+---------+---------+---------+---------+---------+---------+---------|
      MS4  SAAADSTSSALTSRNSTKHTLPNRSNSMKDKSVRYKKFSNAVTSIDSRWPTRRLEFAAQVIVDALKENKAVKPGSGGMTRQDVRDAARIHIGDTGLLDYV
    MS4_h  SAASDSTSSALTSRNSTKHTLPNRSNSMKEKSVRYKKFSSAVTNMDSRWPTRRLEFAAQVIVDALKENKTVKLGSGGMTRQDVRDAARLHIGDTGLLDYV
Consensus  SAAaDSTSSALTSRNSTKHTLPNRSNSMK#KSVRYKKFSnAVTniDSRWPTRRLEFAAQVIVDALKENKaVKlGSGGMTRQDVRDAARiHIGDTGLLDYV

           401    410       420       430       440       450       460       470       480       490       500
           |--------+---------+---------+---------+---------+---------+---------+---------+---------+---------|
      MS4  LKSLNNVIVGNYVVRRMVNPTTRILEYTIHDLGKGLKAPEV-ETEVMAHVDQQVEESSWKPGNDVYCDALFLYKNVLLSYPDSEAVDTAVQTILDSRYFV
    MS4_h  LKSLNNVIIGNYVVRRMVNPSTRILEYTIHDLGKGSKAPEENKPEVMAHADQQVVESSWMLGNDVYSDALFLYKNVLLGYPDSEAVDTAVQTILDSRYFV
Consensus  LKSLNNVI!GNYVVRRMVNPsTRILEYTIHDLGKGlKAPEe.epEVMAHaDQQVeESSWklGNDVYcDALFLYKNVLLgYPDSEAVDTAVQTILDSRYFV

           501    510       520       530       540       550       560       570       580       590       600
           |--------+---------+---------+---------+---------+---------+---------+---------+---------+---------|
      MS4  KEWPVRDEIKEQVLTFICRLQPNFVDKKHELKGVACGEIVVVPLHATVGDLKRASEAALRDTYCIAESLIVTDIKELMDVSDEEVLFGLIQSGVELCVRG
    MS4_h  KEWPVRDEMKEQVLTFICRLQPNFVDKKHELKGLACGEVVVVPLHATVGDLKRAAEAALRDTYCIAERLIVTDIKELMDVSDEEVLFGLIQSGVELCVRG
Consensus  KEWPVRDEiKEQVLTFICRLQPNFVDKKHELKGlACGE!VVVPLHATVGDLKRAaEAALRDTYCIAErLIVTDIKELMDVSDEEVLFGLIQSGVELCVRG

           601    610       620       630       640       650       660       670       680       690       700
           |--------+---------+---------+---------+---------+---------+---------+---------+---------+---------|
      MS4  IAIDLLTPLKYEGGSES-DNWKVRCECGAQDDDGERMVACDICEVWQHTRCCGIDDSETVPPLFVCTGCCDSVLVPSSRTESTVFGVDSADSFLISED-S
    MS4_h  IAIDLLTPLKYQGESESADNWKVRCECGAQDDDGERMVACDICEVWQHTRCYGIDDSETVPPLFVCTGCCDSVLVPSSRTESAAFGVDSVDSFLISEDPT
Consensus  IAIDLLTPLKY#GeSES.DNWKVRCECGAQDDDGERMVACDICEVWQHTRCcGIDDSETVPPLFVCTGCCDSVLVPSSRTESaaFGVDSaDSFLISED.s

           701    710
           |--------|
      MS4  TLLLGYEYGY
    MS4_h  TLLLDYEYGY
Consensus  TLLLdYEYGY
```

Figure S4. Similarity comparison of the soybean MS4 and MS4_HOMOLOG amino acid sequences.

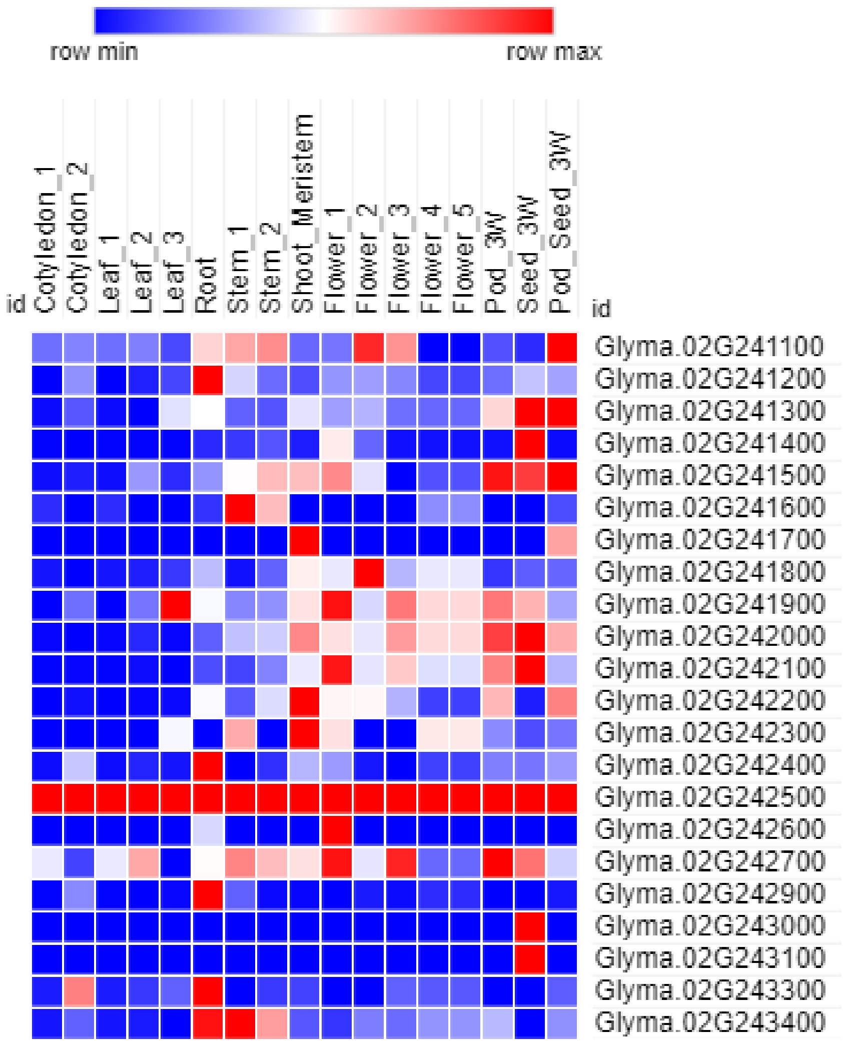

Figure S5. Heatmap representing the expression of the remaining 22 genes present in the mapped region.

(A) Complementation constructs for *GmMS4*

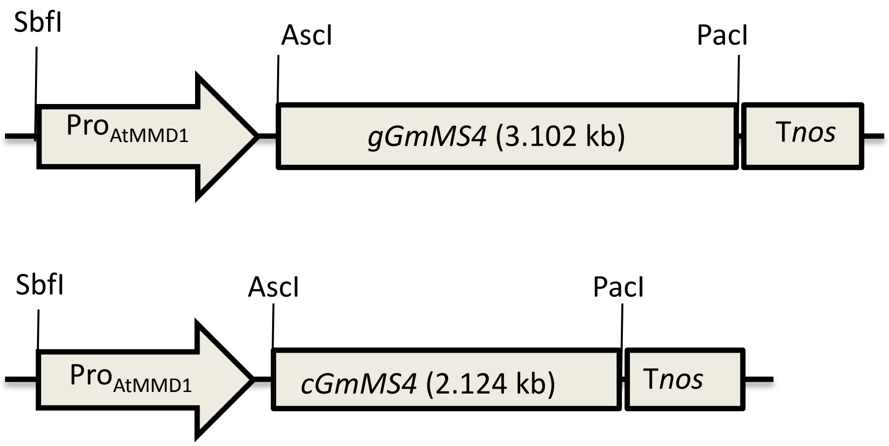

(B) Complementation constructs for *GmMS4_homolog*

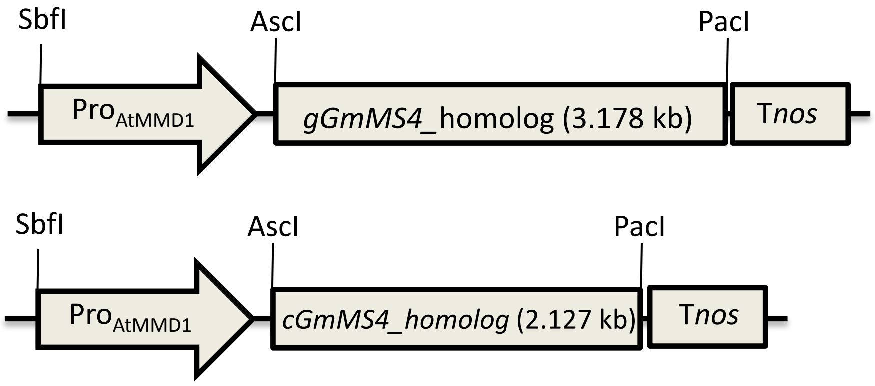

Figure S6. Constructs used for the complementation purposes. **(A)** Arabidopsis *MMD1* native promoter driven genomic and CDS *GmMS4* constructs. **(B)** Arabidopsis *MMD1* native promoter driven genomic and CDS *GmMS4_homolog* constructs.

Table S1. A list of 23 predicted genes in the *ms4* region flanked by BARCSOYSSR_02_1515 and BARCSOYSSR_02_ 1528.

Gene	Start Position	End Position	Predicted Function	Protein Homologs of Interest
BARCSOYSSR_02_1515	42933726	42933751		
Glyma.02G241100	42951620	42957971	UTP--glucose-1-phosphate uridylyltransferase	UTP-glucose-1-phosphate uridylyltra
Glyma.02G241200	42962596	42968168	Isocitrate/isopropylmalate dehydrogenase	NADP-dependent isocitrate dehydrog
Glyma.02G241300	42970608	42978869	ABC1 family	Ubiquinone biosynthesis protein coq-8
Glyma.02G241400	42982745	42989316	Pyridoxal-dependent decarboxylase conserved domain	glutamate decarboxylase/similar to glutamate decarboxylase
Glyma.02G241500	42993453	42995521	Protein kinase domain/Legume lectin domain	L-type lectin-domain receptor kinase/Concanavalin A-like lectin protein
Glyma.02G241600	43010950	43012781	Universal stress protein family	adenine nucleotide alpha hydrolase s/conserved hypothetical protein
Glyma.02G241700	43015662	43017206	-	hypothetical protein
Glyma.02G241800	43022047	43025937	Endomembrane protein 70	transmembrane 9 family protein
Glyma.02G241900	43026406	43032363	PRP38 family	pre-mRNA-splicing factor
Glyma.02G242000	43046963	43048202	Protein of unknown function (DUF775)	Conserved gene of unknown/Uncharacterized protein (80%P)
Glyma.02G242100	43072169	43078866	Myb-like DNA-binding domain	transcription factor KAN2/KANADI like transcription factor
Glyma.02G242200	43083001	43085280	-	transmembrane protein, putative
Glyma.02G242300	43088016	43088773	Fasciclin domain	fasciclin-like arabinogalactan protein/conserved hypothetical protein
Glyma.02G242400	43091693	43094869	EARLY GROWTH RESPONSE PROTEIN-RELATED	similar to Zinc finger protein 4.; [co-ort
Glyma.02G242500	43094090	43094618	-	-
Glyma.02G242600	43103386	43105141	EARLY GROWTH RESPONSE PROTEIN-RELATED	similar to Zinc finger protein 4.; [co-ort
Glyma.02G242700	43112302	43114330	Leucine Rich Repeat/Leucine rich repeat N-terminal domain	receptor protein kinase, putative
Glyma.02G242900	43122457	43123773	U-box domain	Avr9/Cf-9 rapidly elicited protein/similar to U-box domain-containing
Glyma.02G243000	43126795	43128276	B-box zinc finger	PLATZ transcription factor family pr
Glyma.02G243100	43129307	43129745	PLATZ transcription factor	PLATZ transcription factor family pr
Glyma.02G243200	**43132339**	**43135440**	**MIXED-LINEAGE LEUKEMIA 5, MLL5**	**PHD finger protein MALE meiocyte D**
Glyma.02G243300	43137148	43137878	-	-
Glyma.02G243400	43138504	43148118	Cyclic nucleotide-binding domain/Ankyrin repeat	potassium outward rectifying channel
BARCSOYSSR_02_1528	43149577	43149616		

Table S2. Details of primers used in present study for various purposes.

Primer name	Sequence (5'-3')
**A. For amplification of *Glyma.02G243200***	
MS4_F1	GGATTGTTCCCTGGACTAGTTCATTTGTAGTTTTTGTTTGTTTGGG
MS4_F2	AAATTCCAATGACCCTAAAGGAGGGTTAGTATACAACTAAAACTAACCAAGTCCCTT
MS4_F3	AGCAAACAGAAATTCGACCATTCACTTCATTTACTCATTGACTAGTTTCTC
MS4_R1	ATTTGTTGGAGATAGCTTATCTCTTTTTTCCAAAAATAAATTCGATGTGCTATATAGTCCC
MS4_R2	TGCAACTACTCACCTACAACTTTCTCTAATATTTTTGTAACTACCTCATTTCACTTATTTAT CTGATTCTA
MS4_R3	CACTCTCCTCACTCTTTCCCACGTCTTCTCTCATTAGAATTCTCTCTC
**B. For sequencing of *MS4***	
MS4 1F	GGGCCTTGGGCCATTCTTAT
MS4 1R	TTCGGAAACGAACCTACGCA
MS4 2F	TCGTCGCATGGTGAATCCAA
MS4 2R	TCGGCAGAATCCACACCAAA
MS4 3F	CGTGCTCCTGTGCCTAAGAT
MS4 3R	GAGCTCCACACTCACACCTC
MS4 4F	TGCACAAACCTCCGAGTGAG
MS4 4R	TGGATTCACCATGCGACGAA
MS4 5F	GCTGAAAGGTGTAGCATGTGG
MS4 5R	AGATCTAGTCATACACCACTTTGT
MS4 6F	CTACACTGTCTCCGGGAACC
MS4 6R	GGGTTTATGCCACCCATTGT
MS4 7F	AGGGTTGCTTAGTGGTTTGGG
MS4 7R	GTCACTCCAAAGCTTCCACG
MS4 8F	GGCAGGAAGATTGCAGTGGA
MS4 8R	AAATTTGGTTGCAAGCGGCA
MS4 9F	TGTGATTGCGTTTTGTTGCT
MS4 9R	AGGGTTTATGCCACCCATTGT
MS4 10F	TTGAGAAGGGTTGCTTAGTGGT
MS4 10R	ACGGCAAAACCTATAACCCCA
MS4 11F	GGCTCAGGTGGGATGACAAG
MS4 11R	CCGAGTGTGCTGCCATACTT
MS4 12F	AAGCCCCTGAAGTGGAGACT
MS4 12R	AGGGACGTTAACACACCTCAC
**C. For Functional Characterization**	
AtMMD1_pro_F	tcccCCTGCAGGGCAACGGGTGATTACAAAAATTTAAACTTGG
AtMMD1_pro_R	gcttGGCGCGCCCGAATCAGAAATTTGGTTTGATCTTGAGAAG
MS4_AscI_F	gcttGGCGCGCCATGTCTTTCGCTCTCATCGAAGC
MS4_PacI_R	ggccTTAATTAATTAGTATCCATACTCGTATCCTAGTAAAAGGGTAG

Table S3. Duplication gene list of syntenic block 245 between soybean chr02-chr14 with *MS4* and *MS4_homolog* as duplicated gene pair (highlighted in yellow).

BLOCK Number	BLOCK SCORE	E_VALUE	LOCUS_1	LOCUS_2	Ka	Ks
245	9852	2.00E-158	Glyma.02G208200	Glyma.14G176300	0.2327	1.5595
245	9852	0	Glyma.02G208400	Glyma.14G176600	0.021	0.1493
245	9852	0	Glyma.02G208500	Glyma.14G176700	0.0167	0.0787
245	9852	1.00E-159	Glyma.02G208600	Glyma.14G176800	0.0334	0.0943
245	9852	0	Glyma.02G208700	Glyma.14G176900	0.0078	0.1338
245	9852	1.00E-43	Glyma.02G208800	Glyma.14G177000	0.0502	0.2403
245	9852	5.00E-106	Glyma.02G208900	Glyma.14G177100	0.0202	0.0881
245	9852	0	Glyma.02G209000	Glyma.14G177200	0.0206	0.1049
245	9852	0	Glyma.02G209100	Glyma.14G177300	0.0215	0.0943
245	9852	2.00E-81	Glyma.02G209200	Glyma.14G177400	0.0541	0.135
245	9852	0	Glyma.02G209400	Glyma.14G177500	0.0451	0.336
245	9852	7.00E-71	Glyma.02G209500	Glyma.14G177600	0.0831	0.2892
245	9852	0	Glyma.02G209600	Glyma.14G177700	0.0303	0.1387
245	9852	1.00E-62	Glyma.02G210000	Glyma.14G177800	0.2632	0.6246
245	9852	0	Glyma.02G210400	Glyma.14G177900	0.0534	0.1816
245	9852	1.00E-170	Glyma.02G210500	Glyma.14G178000	0.027	0.1415
245	9852	0	Glyma.02G210600	Glyma.14G178200	0.0807	0.1765
245	9852	0	Glyma.02G210800	Glyma.14G178500	0.0499	0.1517
245	9852	0	Glyma.02G211200	Glyma.14G178800	0.0247	0.0817
245	9852	0	Glyma.02G211300	Glyma.14G178900	0.0244	0.0979
245	9852	0	Glyma.02G211400	Glyma.14G179000	0.039	0.1274
245	9852	1.00E-92	Glyma.02G211500	Glyma.14G179100	0.0233	0.1854
245	9852	4.00E-155	Glyma.02G211600	Glyma.14G179200	0.0273	0.145
245	9852	0	Glyma.02G211700	Glyma.14G179300	0.0344	0.151
245	9852	0	Glyma.02G211800	Glyma.14G179500	0.0332	0.1997
245	9852	1.00E-115	Glyma.02G211900	Glyma.14G179600	0.0386	0.17
245	9852	1.00E-99	Glyma.02G212100	Glyma.14G179800	0.029	0.1357
245	9852	0	Glyma.02G212200	Glyma.14G179900	0.0626	0.1481
245	9852	3.00E-34	Glyma.02G212300	Glyma.14G180100	0.0166	0.1487
245	9852	0	Glyma.02G212400	Glyma.14G180300	0.0321	0.1034
245	9852	1.00E-68	Glyma.02G212500	Glyma.14G180400	0.197	0.5054
245	9852	9.00E-141	Glyma.02G212600	Glyma.14G180500	0.0213	0.1298
245	9852	0	Glyma.02G212700	Glyma.14G180600	0.0139	0.0986
245	9852	2.00E-113	Glyma.02G213000	Glyma.14G180700	0.0839	0.3886
245	9852	2.00E-142	Glyma.02G213200	Glyma.14G180800	0.0327	0.2098
245	9852	6.00E-10	Glyma.02G213300	Glyma.14G180900	0.0498	0.2884
245	9852	0	Glyma.02G213400	Glyma.14G181100	0.0502	0.1126
245	9852	1.00E-123	Glyma.02G213500	Glyma.14G181200	0.0961	0.3059
245	9852	0	Glyma.02G213600	Glyma.14G181300	0.0127	0.0965
245	9852	2.00E-93	Glyma.02G213700	Glyma.14G181400	0.0424	0.0839
245	9852	2.00E-118	Glyma.02G213800	Glyma.14G181500	0.0592	0.1642
245	9852	6.00E-168	Glyma.02G213900	Glyma.14G181600	0.0699	0.3069
245	9852	0	Glyma.02G214300	Glyma.14G181800	0.0273	0.1089
245	9852	2.00E-109	Glyma.02G214400	Glyma.14G181900	0.0181	0.133
245	9852	0	Glyma.02G214500	Glyma.14G182000	0.0497	0.1246

245	9852	0	Glyma.02G214700	Glyma.14G182100	0.0477	0.1047
245	9852	0	Glyma.02G215000	Glyma.14G182300	0.0378	0.1362
245	9852	0	Glyma.02G215100	Glyma.14G182400	0.0415	0.1056
245	9852	0	Glyma.02G215200	Glyma.14G182500	0.0149	0.0923
245	9852	0	Glyma.02G215300	Glyma.14G182700	0.0338	0.1532
245	9852	8.00E-180	Glyma.02G215600	Glyma.14G182800	0.0336	0.1071
245	9852	0	Glyma.02G215900	Glyma.14G182900	0.0368	0.0761
245	9852	0	Glyma.02G216000	Glyma.14G183000	0.0231	0.0852
245	9852	2.00E-74	Glyma.02G216100	Glyma.14G183100	0.0082	0.1046
245	9852	4.00E-25	Glyma.02G216200	Glyma.14G183300	0.0187	0.1074
245	9852	5.00E-88	Glyma.02G216500	Glyma.14G183500	0.2046	0.4071
245	9852	1.00E-141	Glyma.02G216600	Glyma.14G183800	0.0315	0.0687
245	9852	6.00E-12	Glyma.02G216800	Glyma.14G184100	0.6474	0.6045
245	9852	0	Glyma.02G216900	Glyma.14G184200	0.0345	0.0835
245	9852	9.00E-43	Glyma.02G217000	Glyma.14G184400	0.0381	0.0731
245	9852	0	Glyma.02G217100	Glyma.14G184500	0.0169	0.098
245	9852	0	Glyma.02G217200	Glyma.14G184600	0.0235	0.0795
245	9852	2.00E-165	Glyma.02G217500	Glyma.14G184700	0.0601	0.1716
245	9852	0	Glyma.02G217800	Glyma.14G185100	0.0234	0.1232
245	9852	5.00E-89	Glyma.02G217900	Glyma.14G185300	0.0384	0.1425
245	9852	0	Glyma.02G218100	Glyma.14G185400	0.0305	0.1384
245	9852	0	Glyma.02G218200	Glyma.14G185600	0.0542	0.2266
245	9852	0	Glyma.02G218300	Glyma.14G185700	0.0102	0.1142
245	9852	6.00E-173	Glyma.02G219000	Glyma.14G186300	0.0429	0.1047
245	9852	7.00E-93	Glyma.02G219100	Glyma.14G186400	0.0902	0.1546
245	9852	0	Glyma.02G219200	Glyma.14G186500	0.1126	0.2157
245	9852	2.00E-159	Glyma.02G219300	Glyma.14G186700	0.1393	0.2227
245	9852	0	Glyma.02G219400	Glyma.14G186800	0.0496	0.1098
245	9852	2.00E-16	Glyma.02G220200	Glyma.14G187600	0.225	0.3758
245	9852	1.00E-93	Glyma.02G220800	Glyma.14G188200	0.0202	0.1679
245	9852	0	Glyma.02G220900	Glyma.14G188400	0.017	0.1243
245	9852	4.00E-105	Glyma.02G221000	Glyma.14G188500	0.0164	0.1313
245	9852	2.00E-111	Glyma.02G221100	Glyma.14G188600	0.0182	0.1586
245	9852	4.00E-172	Glyma.02G221200	Glyma.14G188700	0.0317	0.163
245	9852	4.00E-97	Glyma.02G221300	Glyma.14G188800	0.0403	0.0819
245	9852	2.00E-40	Glyma.02G221700	Glyma.14G189000	0.028	0.1621
245	9852	0	Glyma.02G221900	Glyma.14G189100	0.0223	0.1521
245	9852	0	Glyma.02G222000	Glyma.14G189200	0.0865	0.2872
245	9852	0	Glyma.02G222300	Glyma.14G189300	0.0522	0.1309
245	9852	0	Glyma.02G222400	Glyma.14G189400	0.0227	0.1169
245	9852	5.00E-105	Glyma.02G222600	Glyma.14G189500	0.0242	0.1354
245	9852	2.00E-53	Glyma.02G222700	Glyma.14G189600	0.079	0.141
245	9852	9.00E-23	Glyma.02G222800	Glyma.14G189700	0.0901	0.3757
245	9852	0	Glyma.02G222900	Glyma.14G189900	0.0228	0.0701
245	9852	0	Glyma.02G223100	Glyma.14G190000	0.0114	0.0998
245	9852	0	Glyma.02G223300	Glyma.14G190100	0.0212	0.134
245	9852	0	Glyma.02G223400	Glyma.14G190200	0.0191	0.0753

245	9852	4.00E-92	Glyma.02G223600	Glyma.14G190300	0.0203	0.2029
245	9852	0	Glyma.02G223700	Glyma.14G190400	0.0245	0.0929
245	9852	0	Glyma.02G223800	Glyma.14G190500	0.0551	0.1291
245	9852	0	Glyma.02G223900	Glyma.14G190600	0.0198	0.1262
245	9852	0	Glyma.02G224000	Glyma.14G190700	0.0212	0.1072
245	9852	1.00E-53	Glyma.02G224100	Glyma.14G190800	0	0.5871
245	9852	0	Glyma.02G224200	Glyma.14G190900	0.0293	0.1375
245	9852	2.00E-50	Glyma.02G224300	Glyma.14G191000	0.0771	0.133
245	9852	0	Glyma.02G224400	Glyma.14G191100	0.0503	0.1778
245	9852	0	Glyma.02G224600	Glyma.14G191300	0.0388	0.1669
245	9852	2.00E-113	Glyma.02G224700	Glyma.14G191400	0.0944	0.2085
245	9852	0	Glyma.02G224800	Glyma.14G191500	0.0099	0.1402
245	9852	0	Glyma.02G224900	Glyma.14G191700	0.0234	0.1175
245	9852	0	Glyma.02G225000	Glyma.14G191900	0.041	0.1096
245	9852	0	Glyma.02G225200	Glyma.14G192000	0.0729	0.1042
245	9852	0	Glyma.02G225300	Glyma.14G192100	0.0156	0.0813
245	9852	0	Glyma.02G225400	Glyma.14G192200	0.0444	0.1331
245	9852	2.00E-128	Glyma.02G225500	Glyma.14G192300	0.0287	0.1242
245	9852	9.00E-65	Glyma.02G225600	Glyma.14G192400	0.0407	0.0965
245	9852	0	Glyma.02G225700	Glyma.14G192600	0.1009	0.1963
245	9852	0	Glyma.02G225800	Glyma.14G192800	0.0907	0.2349
245	9852	0	Glyma.02G226100	Glyma.14G192900	0.0538	0.1037
245	9852	0	Glyma.02G226200	Glyma.14G193000	0.0592	0.2063
245	9852	0	Glyma.02G226300	Glyma.14G193200	0.0209	0.1096
245	9852	9.00E-124	Glyma.02G226400	Glyma.14G193300	0.0438	0.1018
245	9852	0	Glyma.02G226500	Glyma.14G193400	0.0207	0.1193
245	9852	0	Glyma.02G226600	Glyma.14G193500	0.0339	0.1829
245	9852	0	Glyma.02G226700	Glyma.14G193600	0.0347	0.1111
245	9852	1.00E-135	Glyma.02G226800	Glyma.14G193800	0.0428	0.1198
245	9852	0	Glyma.02G226900	Glyma.14G193900	0.0337	0.1564
245	9852	0	Glyma.02G227000	Glyma.14G194100	0.0355	0.1273
245	9852	0	Glyma.02G227100	Glyma.14G194200	0.0736	0.1532
245	9852	0	Glyma.02G227200	Glyma.14G194300	0.0232	0.1337
245	9852	0	Glyma.02G227300	Glyma.14G194400	0.0242	0.1144
245	9852	2.00E-108	Glyma.02G227600	Glyma.14G194500	0.0292	0.1466
245	9852	0	Glyma.02G227700	Glyma.14G194600	0.0189	0.0809
245	9852	0	Glyma.02G227900	Glyma.14G194800	0.0601	0.1345
245	9852	0	Glyma.02G228000	Glyma.14G194900	0.0465	0.0877
245	9852	0	Glyma.02G228100	Glyma.14G195000	0.0082	0.0791
245	9852	0	Glyma.02G228200	Glyma.14G195200	0.0187	0.1155
245	9852	0	Glyma.02G228300	Glyma.14G195300	0.078	0.1625
245	9852	0	Glyma.02G228400	Glyma.14G195400	0.007	0.0962
245	9852	0	Glyma.02G228600	Glyma.14G195600	0.035	0.0942
245	9852	0	Glyma.02G228700	Glyma.14G195700	0.0182	0.0898
245	9852	3.00E-107	Glyma.02G228800	Glyma.14G195900	0.0634	0.1921
245	9852	8.00E-62	Glyma.02G228900	Glyma.14G196000	0.1249	0.2429
245	9852	0	Glyma.02G229700	Glyma.14G196800	0.0157	0.1121

245	9852	4.00E-88	Glyma.02G230200	Glyma.14G197300	0.0407	0.3785
245	9852	0	Glyma.02G230300	Glyma.14G197400	0.07	0.1635
245	9852	5.00E-81	Glyma.02G230400	Glyma.14G197500	0.0617	0.1346
245	9852	0	Glyma.02G230500	Glyma.14G197600	0.0055	0.1234
245	9852	1.00E-90	Glyma.02G230600	Glyma.14G197700	0.091	0.1722
245	9852	7.00E-173	Glyma.02G230700	Glyma.14G197800	0.0464	0.1101
245	9852	1.00E-121	Glyma.02G230800	Glyma.14G197900	0.0723	0.1688
245	9852	2.00E-172	Glyma.02G230900	Glyma.14G198000	0.4218	1.6266
245	9852	0	Glyma.02G231100	Glyma.14G198700	0.0287	0.0937
245	9852	0	Glyma.02G231200	Glyma.14G198800	0.0178	0.2107
245	9852	0	Glyma.02G231600	Glyma.14G198900	0.0116	0.2037
245	9852	0	Glyma.02G231800	Glyma.14G199000	0.0334	0.1654
245	9852	0	Glyma.02G232100	Glyma.14G199200	0.0347	0.2134
245	9852	2.00E-78	Glyma.02G232500	Glyma.14G199900	0.1094	0.249
245	9852	0	Glyma.02G232600	Glyma.14G200200	0.0223	0.1245
245	9852	0	Glyma.02G232700	Glyma.14G200300	0.0699	0.2075
245	9852	2.00E-153	Glyma.02G232800	Glyma.14G200400	0.0111	0.1046
245	9852	1.00E-119	Glyma.02G233000	Glyma.14G200600	0.0338	0.1101
245	9852	7.00E-119	Glyma.02G233100	Glyma.14G200700	0.0465	0.1498
245	9852	0	Glyma.02G233200	Glyma.14G201200	0.0479	0.1589
245	9852	3.00E-101	Glyma.02G233300	Glyma.14G201300	0.045	0.1313
245	9852	0	Glyma.02G233700	Glyma.14G201500	0.0204	0.104
245	9852	0	Glyma.02G233800	Glyma.14G201600	0.0281	0.1479
245	9852	3.00E-172	Glyma.02G233900	Glyma.14G201700	0.1823	0.7166
245	9852	0	Glyma.02G234200	Glyma.14G201800	0.0275	0.1027
245	9852	2.00E-150	Glyma.02G234300	Glyma.14G202000	0.0388	0.1586
245	9852	0	Glyma.02G234500	Glyma.14G202100	0.0286	0.1798
245	9852	2.00E-109	Glyma.02G234600	Glyma.14G202300	0.0319	0.0878
245	9852	6.00E-35	Glyma.02G234800	Glyma.14G202700	0.0732	0.2938
245	9852	3.00E-50	Glyma.02G234900	Glyma.14G202800	0.0946	0.146
245	9852	9.00E-38	Glyma.02G235000	Glyma.14G202900	0.229	0.3794
245	9852	0	Glyma.02G235100	Glyma.14G203000	0.0244	0.1955
245	9852	0	Glyma.02G235400	Glyma.14G203400	0.038	0.1343
245	9852	8.00E-103	Glyma.02G235500	Glyma.14G203500	0.0516	0.236
245	9852	0	Glyma.02G235600	Glyma.14G203600	0.0358	0.1292
245	9852	0	Glyma.02G235700	Glyma.14G203700	0.023	0.118
245	9852	0	Glyma.02G235800	Glyma.14G203800	0.0352	0.0931
245	9852	6.00E-167	Glyma.02G235900	Glyma.14G203900	0.012	0.0969
245	9852	6.00E-73	Glyma.02G236100	Glyma.14G204000	0.0912	0.1913
245	9852	0	Glyma.02G236200	Glyma.14G204100	0.0285	0.1458
245	9852	3.00E-157	Glyma.02G236300	Glyma.14G204200	0.0252	0.1692
245	9852	0	Glyma.02G236400	Glyma.14G204400	0.051	0.1181
245	9852	0	Glyma.02G236600	Glyma.14G205100	0.032	0.1175
245	9852	0	Glyma.02G236700	Glyma.14G205500	0.0147	0.062
245	9852	7.00E-134	Glyma.02G236800	Glyma.14G205600	0.0545	0.2289
245	9852	8.00E-110	Glyma.02G236900	Glyma.14G205700	0	0.1413
245	9852	0	Glyma.02G237000	Glyma.14G206000	0.0265	0.1771

245	9852	0	Glyma.02G237100	Glyma.14G206100	0.0293	0.1521
245	9852	4.00E-112	Glyma.02G237200	Glyma.14G206200	0.136	0.2773
245	9852	2.00E-74	Glyma.02G237400	Glyma.14G206500	0.2308	0.2795
245	9852	0	Glyma.02G237600	Glyma.14G206600	0.1002	0.1609
245	9852	3.00E-36	Glyma.02G237700	Glyma.14G206700	0.0975	0.337
245	9852	0	Glyma.02G237800	Glyma.14G206800	0.0304	0.107
245	9852	0	Glyma.02G238300	Glyma.14G207200	0.0144	0.1318
245	9852	0	Glyma.02G238400	Glyma.14G207300	0.0115	0.0663
245	9852	0	Glyma.02G238500	Glyma.14G207400	0.0627	0.1459
245	9852	0	Glyma.02G238600	Glyma.14G207500	0.0306	0.1474
245	9852	7.00E-148	Glyma.02G238700	Glyma.14G207600	0.0162	0.1619
245	9852	0	Glyma.02G238800	Glyma.14G207700	0.0183	0.2137
245	9852	0	Glyma.02G239000	Glyma.14G207900	0.0462	0.1388
245	9852	1.00E-120	Glyma.02G239100	Glyma.14G208000	0.0249	0.1508
245	9852	0	Glyma.02G239200	Glyma.14G208100	0.0411	0.0777
245	9852	0	Glyma.02G239300	Glyma.14G208200	0.0384	0.3072
245	9852	0	Glyma.02G239600	Glyma.14G208500	0.0104	0.1075
245	9852	1.00E-51	Glyma.02G239700	Glyma.14G208600	0	0.1804
245	9852	7.00E-72	Glyma.02G239800	Glyma.14G209000	0.0651	0.109
245	9852	1.00E-174	Glyma.02G239900	Glyma.14G209200	0.0767	0.1347
245	9852	0	Glyma.02G240000	Glyma.14G209400	0.0278	0.1259
245	9852	0	Glyma.02G240200	Glyma.14G209700	0.0616	0.1453
245	9852	0	Glyma.02G240300	Glyma.14G209800	0.064	0.1485
245	9852	0	Glyma.02G240400	Glyma.14G209900	0.0113	0.0986
245	9852	0	Glyma.02G240500	Glyma.14G210000	0.0331	0.1378
245	9852	6.00E-162	Glyma.02G240600	Glyma.14G210100	0.0058	0.1489
245	9852	0	Glyma.02G240700	Glyma.14G210200	0.0238	0.0941
245	9852	0	Glyma.02G240800	Glyma.14G210400	0.0191	0.1521
245	9852	1.00E-177	Glyma.02G240900	Glyma.14G210500	0.0187	0.1855
245	9852	0	Glyma.02G241000	Glyma.14G210600	0.0455	0.1291
245	9852	0	Glyma.02G241100	Glyma.14G210700	0.0302	0.1441
245	9852	0	Glyma.02G241200	Glyma.14G211000	0.0031	0.1103
245	9852	0	Glyma.02G241400	Glyma.14G211100	0.0174	0.1159
245	9852	0	Glyma.02G241500	Glyma.14G211200	0.024	0.181
245	9852	1.00E-95	Glyma.02G241600	Glyma.14G211300	0.0427	0.1028
245	9852	7.00E-125	Glyma.02G241700	Glyma.14G211400	0.056	0.0869
245	9852	0	Glyma.02G241800	Glyma.14G211500	0.0262	0.1132
245	9852	8.00E-177	Glyma.02G241900	Glyma.14G211600	0.0228	0.1209
245	9852	9.00E-137	Glyma.02G242000	Glyma.14G211800	0.0178	0.0861
245	9852	0	Glyma.02G242100	Glyma.14G211900	0.0461	0.1447
245	9852	1.00E-171	Glyma.02G242400	Glyma.14G212000	0.0153	0.1174
245	9852	0	Glyma.02G242700	Glyma.14G212100	0.0761	0.2238
245	9852	0	Glyma.02G242900	Glyma.14G212200	0.0366	0.1749
245	9852	0	Glyma.02G243200	Glyma.14G212300	0.0357	0.0986
245	9852	1.00E-25	Glyma.02G243300	Glyma.14G212400	0.0751	0.1301
245	9852	0	Glyma.02G243400	Glyma.14G212500	0.0242	0.1019
245	9852	0	Glyma.02G243500	Glyma.14G212600	0.0469	0.1235

245	9852	0	Glyma.02G243600	Glyma.14G212700	0.0375	0.209
245	9852	0	Glyma.02G243800	Glyma.14G212900	0.04	0.0752
245	9852	1.00E-47	Glyma.02G243900	Glyma.14G213200	1.1086	1.2322
245	9852	5.00E-136	Glyma.02G244100	Glyma.14G213400	0.016	0.0906
245	9852	1.00E-158	Glyma.02G244200	Glyma.14G213600	0.0408	0.1946
245	9852	0	Glyma.02G244700	Glyma.14G214300	0.0342	0.1659
245	9852	2.00E-63	Glyma.02G245000	Glyma.14G215100	0.0366	0.2114
245	9852	0	Glyma.02G245200	Glyma.14G215300	0.0426	0.1191
245	9852	0	Glyma.02G245300	Glyma.14G215400	0.016	0.0535
245	9852	0	Glyma.02G245400	Glyma.14G215500	0.0189	0.1113
245	9852	2.00E-66	Glyma.02G245600	Glyma.14G215600	0.0136	0.0776
245	9852	5.00E-93	Glyma.02G245700	Glyma.14G215800	0.0257	0.2063
245	9852	2.00E-158	Glyma.02G245800	Glyma.14G215900	0.0252	0.08
245	9852	4.00E-54	Glyma.02G245900	Glyma.14G216000	0.0262	0.1122
245	9852	0	Glyma.02G246000	Glyma.14G216100	0.0331	0.1187

Table S4. List of SRA files used for the expression analysis of *GmMS4* and *GmMS4_h*.

**NCBI Accessions**	**Sample Name**	**Description**
SRR1174214	Cotyledon_1	Germination stage
SRR1174228	Cotyledon_2	Trefoil stage
SRR1174226	Leaf_1	Trefoil stage
SRR1174229	Leaf_2	Flower bud differentiation stage
SRR1174206	Leaf_3	Senescent leaves
SRR1174205	Root	Germination stage
SRR1174213	Stem_1	Germination stage
SRR1174216	Stem_2	Trefoil stage
SRR1174231	Shoot_meristem	Flower bud differentiation stage
SRR1174232	Flower_1	Flower bud differentiation stage
SRR1174217	Flower_2	Flowering stage: bud before flowering
SRR1174218	Flower_3	Flowering stage: florescence
SRR1174220	Flower_4	Flowering stage: 5 d after flowering
SRR1174219	Flower_5	Flowering stage: florescence: different stage
SRR1174221	Pod_3W	3 weeks old pod
SRR1174211	Pod_seed_3W	3 weeks old pod_seed
SRR1174222	Seed__3W	3 weeks old seed
